# Revisiting gastric cancer disparities in Asian American subgroups: insights from the SEER database

**DOI:** 10.3389/fonc.2026.1858617

**Published:** 2026-06-16

**Authors:** Peng Luo, Xiaoxiao Fan, Guojun Chen, Kaiyang Zhu, Linghua Chen, Yuzhi (Michael) Bai, Shihua Wang, Linghua Zhu

**Affiliations:** 1 Department of Gastrointestinal Surgery, Sir Run Run Shaw Hospital, Zhejiang University School of Medicine, Hangzhou, China; 2 Advanced Micro Devices, Inc (AMD) Software Develop Central, Santa Clara, CA, United States; 3 Shanghai American School, Shanghai, China; 4 The Ohio State University Comprehensive Cancer Center, Columbus, OH, United States

**Keywords:** Asian American, gastric cancer, heterogeneity, stage, survival

## Abstract

**Background:**

Gastric cancer disproportionately affects Asian Americans and exhibits substantial heterogeneity across ethnic subgroups.

**Methods:**

Patients with gastric cancer were identified from the Surveillance, Epidemiology, and End Results (SEER) database (2000-2021).

**Results:**

A total of 16,321 Asian American patients were obtained, with 29% diagnosed at a localized stage. South Asian patients were more likely to be younger, male, married, have cardia tumors, present with low-grade tumors, and receive chemotherapy, and exhibited the most favorable 5-year cancer-specific and overall survival rates across the entire cohort and most subgroups. From 2000–2010 to 2011-2021, survival did not significantly improve among Chinese and Japanese patients, but improved in other Asian American subgroups, as well as White and Black patients, with the greatest gains observed in South Asians. In the most recent decade, South Asians had the highest survival rates, whereas Japanese patients had the lowest, with White and Black patients surpassing Japanese patients. Despite these disparities, clinicopathological trends were largely consistent across subgroups, including increases in localized-stage diagnosis, cardia tumors, and chemotherapy use, alongside decreased use of surgery and radiotherapy.

**Conclusions:**

Substantial heterogeneity persists in clinicopathological features, survival, and temporal trends among Asian American subgroups, with South Asian now demonstrating the most favorable survival outcomes and consistent increases in early diagnosis, cardia tumors, and systemic therapy use. These findings help identify factors underlying this heterogeneity and develop strategies to reduce incidence, improve early detection, and mitigate survival disparities.

## Introduction

Asian Americans represent a highly diverse population characterized by significant social, economic, and cultural heterogeneity, originating from over 20 different countries in the Far East, Southeast Asia, and the Indian subcontinent. As of 2023, approximately 28 million Asian Americans reside in the US, comprising about 7.4% of the total population (1). The six largest Asian-origin subgroups in the US are Chinese (21%), Asian Indian (20%), Filipino (18%), Vietnamese (9%), Korean (8%), and Japanese (7%) (1). Asian Americans are the fastest-growing demographic, with a 109% increase since 2000 - primarily driven by international migration. From 2000 to 2023, the growth rates among Asian American subgroups varied considerably: Japanese Americans increased by 36%, Koreans by 56%, Vietnamese by 83%, Filipinos by 89%, Chinese by 95%, and Indian Americans by an impressive 174% (1). This dynamic and diverse growth of the Asian American population highlights the need to address health disparities, such as gastric cancer, across distinct ethnic subgroups.

Gastric cancer is the fifth most common cancer and the third leading cause of cancer-related deaths worldwide, with the highest incidence and mortality rates found in Eastern Asian countries (2). Although the overall incidence and mortality rates of gastric cancer are low in the US, significant racial/ethnic disparities persist (3, 4). Between 2017 and 2021, the incidence of gastric cancer among Asian Americans was 8.9 per 100,000 population compared to 5.2 among White individuals. Additionally, Asian Americans are approximately twice as likely to die from stomach cancer, with mortality rates of 4.2 per 100,000 in contrast to 2.0 for White people (5). The aggregated data for Asian Americans conceals larger variations among specific ethnic subgroups. Among individuals aged 50 years or older, incidence rates of non-cardia gastric cancer were notably higher compared to their White counterparts: Koreans had a 13.3-fold higher incidence, Vietnamese 6.5-fold, Chinese 4.8-fold, Japanese 5.2-fold, South Asians 2.1-fold, and Filipino Americans 1.8-fold (6). Furthermore, gastric cancer accounts for up to 15% of cancer deaths among Chinese, Korean, and Vietnamese Americans, in contrast to less than 2% among non-Hispanic White people (7). Despite facing heavier burdens of gastric cancer at the population level, Asian American patients have more favorable survival rates than White patients (8–12). These pronounced disparities in incidence, mortality, and outcomes further emphasize the necessity for research on gastric cancer within disaggregated Asian American ethnic subgroups.

Asian Americans with gastric cancer demonstrated diverse clinicopathological features and variable survival outcomes across ethnic subgroups (11, 13, 14). Korean patients consistently exhibited better 5-year survival rates compared to other Asian American subgroups (11, 13, 14). However, after adjusting for relevant prognostic factors, the survival outcomes were largely comparable among Koreans and five other major Asian American ethnic subgroups with gastric cancer (11). It is important to note that most prior studies on gastric cancer among Asian Americans have relied on data that is more than a decade old. This present study seeks to utilize an updated national cancer database to better characterize gastric cancer and assess survival disparities among Asian Americans. In addition, White and Black patients with gastric cancer were included as reference subgroups to provide a broader context for comparison.

## Materials and methods

Patients diagnosed with gastric cancer were obtained from the SEER 17 registries 2000-2021 (15). Based on the variable “Race_ethnicity”, Asian Americans were classified into the following ethnic subgroups: Asian Indian, Chinese, Filipino, Hmong, Japanese, Kampuchean, Korean, Laotian, Pakistani, Thai, and Vietnamese, and other Asians with unknown ethnicity. We combined Asian Indians and Pakistanis into a single South Asian subgroup. This study focused on the six largest US Asian ethnic subgroups, each having at least 1,000 patients. In addition, White and Black patients were also included for comparative reference. Patients with unknown survival time or unknown median household income inflation-adjusted to 2022 (referred to as “income” hereafter) were excluded from the study. Detailed information about gastric cancer patients by races/ethnicities is presented the **Supplementary Table 1**. Survival time was calculated in months from the date of diagnosis to the date of death or the last alive follow-up date before December 31, 2021. Human investigation approval was not required as the data are from a de-identified public database.

Other covariates, including age, sex, marital status, and income, were obtained from the database. Anatomic sites were divided into cardia (C16.0), fundus, body (C16.2), lesser curvatures (C16.5), greater curvatures (C16.6), antrum (C16.3), pylorus (C16.4), and overlapping or not otherwise specified (NOS) (C16.8 and C16.9). Histological types were classified according to Lauren’s classification and prior studies, including diffuse type (codes 8020–8022, 8142, 8145, and 8490), intestinal type (8140, 8144, 8210–8211, 8260, and 8480–8481), and others (11, 16). Additional clinical covariates extracted from the database included the SEER combined summary stage at diagnosis (localized, regional, distant, and unknown) (11), tumor grade (I–IV and unknown), tumor size, and treatment modality (surgery, chemotherapy, and radiation).

### Statistical analysis

Continuous variables are presented as means ± standard deviations and were analyzed using ANOVA, followed by group-wise comparisons with the Tukey test. Categorical variables were shown as frequencies and percentages and were assessed by the Chi-square test. Survival curves were generated, and 5-year survival rates were calculated via the Kaplan-Meier method, with differences evaluated using the Log-Rank test. To evaluate the temporal change in survival between the 2000–2010 and 2011–2021 periods, patients who survived over 120 months were censored at 120 months. Univariable and multivariable Cox proportional hazards regression models were also employed to estimate hazard ratios (HRs) and corresponding 95% confidence intervals (CIs) for both cancer-specific and overall mortality. All data analyses were performed using SAS 9.4 (Raleigh, NC), and a two-sided P value <0.05 was considered to be statistically significant.

## Results

### Overall characteristics of Asian American patients with gastric cancer diagnosed between 2000 and 2021

We identified a total of 16,321 Asian Americans with gastric cancer. Among these patients, 53% were diagnosed at 70 years or older. The cohort comprised 57% males and 87% residing in the West region of the US. The most frequent anatomical sites of gastric cancer were the antrum (28%), followed by the cardia (13%). Based on the SEER combined summary stage, 29% of patients were diagnosed at the localized stage. Histologically, 24% of tumors were identified as diffuse type, whereas 59% were intestinal type. Additionally, 56% of patients underwent surgery, 42% received chemotherapy, and 19% were treated with radiotherapy (**Table 1**).

**Table 1:** Characteristics of Asian American patients with gastric cancer diagnosed during 2000–2021.

Variable	All Asians n (%)	Chinese n (%)		Korean n (%)	Japanese n (%)	Filipino n (%)	Vietnamese n (%)	South Asian n (%)	P value^#^	White n (%)	Black n (%)	P value^##^
	16,321 (100)	4,340 (27)		3,787 (23)	3,207 (20)	2,173 (13)	1,775 (11)	1,039 (6)		97,407 (74)	18,065 (14)	
Age (year)												
Mean ± SD	68.9 ± 13.9	70 ± 14^a^		67 ± 13^b^	75 ± 12^c^	68 ± 14^b,d^	66 ± 15^d^	62 ± 15^e^	P<0.0001	67.8 ± 14.0	65.9 ± 13.8^c^	P<0.0001
Group									P<0.0001			P<0.0001
<50	1,651 (10)	439 (10)		394 (10)	114 (4)	205 (9)	270 (15)	229 (22.0)		10,537 (11)	2,250 (12)	
50- 59	2,277 (14)	546 (13)		608 (16)	256 (8)	346 (16)	310 (17)	211 (20.3)		15,385 (16)	3,443 (19)	
60-69	3,682 (23)	941 (22)		1,001 (26)	505 (16)	561 (26)	413 (23)	261 (25.1)		23,471 (24)	4,729 (26)	
70-79	4,604 (28)	1,190 (27)		1,103 (29)	1,020 (32)	629 (29)	442 (25)	220 (21.2)		26,015 (27)	4,406 (24)	
≥80	4,107 (25)	1,224 (28)		681 (18)	1,312 (41)	432 (20)	340 (19)	118 (11)		21,999 (23)	3,237 (18)	
Gender									P<0.0001			P<0.0001
Male	9,221 (57)	2,439 (56)		2,231 (59)	1,725 (54)	1124 (52)	1057 (60)	645 (62)		60267 (62)	10,179 (56)	
Female	7,100 (44)	1,901 (44)		1,556 (41)	1,482 (46)	1,049 (48)	718 (40)	394 (38)		37,140 (38)	7,886 (44)	
Marital status									P<0.0001			P<0.0001
Married	10,672 (65)	2,994 (69)		2,546 (67)	1,850 (58)	1,361 (63)	1,179 (66)	742 (71)		55,245 (57)	6,899 (38)	
Unmarried	4,931 (30)	1,178 (27)		1,043 (28)	1,231 (38)	737 (34)	511 (29)	231 (22)		36,915 (38)	9,976 (55)	
Unknown	718 (4)	168 (4)		198 (5)	126 (4)	75 (3)	85 (5)	66 (6)		5,247 (5)	1,190 (7)	
Region									P<0.0001			P<0.0001
West	14,200 (87)	3,866 (89)		3,088 (82)	3,101 (97)	2,007 (92)	1,587 (89)	551 (53)		55,449 (57)	5,204 (29)	
South	651 (4)	113 (3)		242 (6)	45 (1)	17 (1)	113 (6)	121 (12)		18,058 (19)	9,153 (51)	
Midwest	53 (0)	7 (0)		5 (0)	10 (0)	3 (0)	22 (1)	6 (1)		3,835 (4)	120 (1)	
Northeast	1,417 (9)	354 (8)		452 (12)	51 (2)	146 (7)	53 (3)	361 (35)		20,065 (21)	3,588 (20)	
Income									P<0.0001			P<0.0001
<75,000	1,305 (8)	268 (6)		278 (7)	309 (10)	169 (8)	156 (9)	125 (12)		28,137 (29)	7,892 (44)	
75,000-94,999	6,032 (37)	1,441 (33)		,1895 (50)	985 (31)	852 (39)	546 (31)	313 (30)		36,816 (38)	6,202 (34)	
≥ 95,000	8,984 (55)	2,631 (61)		1,614 (43)	1,913 (60)	1,152 (53)	1,073 (60)	601 (58)		32,454 (33)	3,971 (22)	
Primary site									P<0.0001			P<0.0001
Antrum	4,489 (28)	1,275 (29)		1,244 (33)	850 (27)	401 (18)	558 (31)	161 (16)		13,701 (14)	4,024 (22)	
Body	1,950 (12)	439 (10)		497 (13)	443 (14)	234 (11)	181 (10)	156 (15)		9,854 (10)	2,094 (12)	
Cardia	2,054 (13)	528 (12)		219 (6)	454 (14)	450 (21)	170 (10)	233 (22)		32,766 (34)	2,297 (13)	
Fundus	665 (4)	203 (5)		116 (3)	105 (3)	127 (6)	56 (3)	58 (6)		4,864 (5)	1,060 (6)	
Greater curvature	821 (5)	229 (5)		154 (4)	145 (5)	147 (7)	97 (5)	49 (5)		3,900 (4)	967 (5)	
Lesser curvature	1,888 (12)	514 (12)		526 (14)	371 (12)	193 (9)	200 (11)	84 (8)		6,155 (6)	1,602 (9)	
Pylorus	513 (3)	150 (3)		128 (3)	74 (2)	55 (3)	84 (5)	22 (2)		2,137 (2)	644 (4)	
Overlapping or not specified	3,941 (24)	1002 (23)		903 (24)	765 (24)	566 (26)	429 (24)	276 (27)		24,030 (25)	5,377 (30)	
Size (cm)									P<0.0001			P<0.0001
≤ 3	3,353 (21)	826 (19)		983 (26)	577 (18)	375 (17)	345 (19)	247 (24)		24,069 (18)	2,943 (16)	
>3	6,418 (39)	1,777 (41)		1,321 (35)	1,306 (41)	931 (43)	750 (42)	333 (32)		45,402 (34)	6,840 (38)	
Unknown	6,550 (40)	1,737 (40)		1,483 (39)	1,324 (41)	867 (40)	680 (38)	459 (44)		62,322 (47)	8,282 (46)	
Stage									P<0.0001			P<0.0001
Localized	4,709 (29)	1,129 (26)		1,299 (34)	936 (29)	587 (27)	418 (24)	340 (33)		27,368 (28)	5,544 (31)	
Regional	4,915 (30)	1,363 (31)		1180 (31)	965 (30)	585 (27)	570 (32)	252 (24)		24,334 (25)	4,373 (24)	
Distant	5,048 (31)	1,344 (31)		956 (25)	956 (30)	819 (38)	636 (36)	337 (32)		35,016 (36)	6,119 (34)	
Unknown	1,649 (10)	504 (12)		352 (9)	350 (11)	182 (8)	151 (9)	110 (11)		10,689 (11)	2,029 (11)	
Histology									P<0.0001			P<0.0001
Diffuse	3,994 (24)	1,032 (24)		995 (26)	720 (22)	563 (26)	476 (27)	208 (20)		19,189 (20)	2,948 (16)	
Intestinal	9,570 (59)	2,592 (60)		2350 (62)	2,020 (63)	1,098 (51)	1,030 (58)	480 (46)		57,048 (59)	10,171 (56)	
Other	2,757 (17)	716 (17)		442 (12)	467 (15)	512 (24)	269 (15)	351 (34)		21,170 (22)	4,946 (27)	
Grade									P<0.0001			P<0.0001
I	961 (6)	232 (5)		192 (5)	193 (6)	129 (6)	74 (4)	141 (14)		7,879 (8)	1,739 (10)	
II	3,271 (20)	848 (20)		807 (21)	711 (22)	389 (18)	344 (19)	172 (17)		19,548 (20)	3,701 (20)	
III/IV	9,121 (56)	2,433 (56)		2,201 (58)	1,798 (56)	1,155 (53)	1,058 (60)	476 (46)		4,7492 (49)	7,952 (44)	
Unknown	2,968 (18)	827 (19)		587 (16)	505 (16)	500 (23)	299 (17)	250 (24)		22,488 (23)	4,673 (26)	
Surgery									P<0.0001			P<0.0001
No	7,239 (44)	1,937 (45)		1,409 (37)	1,455 (45)	1,118 (51)	811 (46)	509 (49)		54,274 (56)	9,652 (53)	
Yes	9,082 (56)	2,403 (55)		2,378 (63)	1,752 (55)	1,055 (49)	964 (54)	530 (51)		43,133 (44)	8,413 (47)	
Chemotherapy									P<0.0001			P<0.0001
No	9,497 (58)	,2479 (57)		2,302 (61)	2,068 (64)	1,154 (53)	958 (54)	536 (52)		54,865 (56)	10,956 (61)	
Yes	6,824 (42)	1,861 (43)		1,485 (39)	1,139 (36)	1,019 (47)	817 (46)	503 (48)		42,542 (44)	7,109 (39)	
Radiotherapy									P<0.0001			P<0.0001
No	13,179 (81)	3,504 (81)		3,132 (83)	2,562 (80)	1,740 (80)	1,411 (79)	830 (80)		76,041 (78)	15,167 (84)	
Yes	3,142 (19)	836 (19)		655 (17)	645 (20)	433 (20)	364 (21)	209 (20)		21,366 (22)	2,898 (16)	
Cancer specific death									P<0.0001			P<0.0001
No	7,487 (46)	1,961 (45)		1,931 (51)	1,258 (39)	968 (45)	765 (43)	604 (58)		38,709 (40)	7,785 (43)	
Yes	8,834 (54)	2,379 (55)		1,856 (49)	1,949 (61)	1,205 (55)	1,010 (57)	435 (42)		58,698 (60)	10,280 (57)	
Overall death									P<0.0001			P<0.0001
No	4,828 (30)	1,306 (30)		1,338 (35)	565 (18)	610 (28)	524 (30)	485 (47)		23,083 (24)	4,509 (25)	
Yes	11,493 (70)	3,034 (70)		2,449 (65)	2,642 (82)	1,563 (72)	1,251 (70)	554 (53)		74,324 (76)	13,556 (75)	

**#** indicates the P value for comparison among Asian American ethnic groups;.

**##** indicates the P value for comparison among white, black, and overall Asian Americans; Mean ages followed by different superscript letters indicate statistically significant differences.

### Comparison of characteristics among Asian American, White, and Black patients with gastric cancer diagnosed between 2000 and 2021

Among Asian American patients, South Asians were diagnosed with gastric cancer at the youngest average age (61.6 years). This subgroup also had the highest proportions of male patients (62%), married individuals (71%), residents of the Northeast region (35%), tumors located in the gastric cardia (22%), and grade I tumors (14%). Notably, South Asians had the lowest proportion (20%) of the diffuse histological type (**Table 1**). Koreans exhibited the highest proportions of localized-stage cancer (34%), tumors in the antrum of the stomach (33%), and receipt of surgical treatment (63%). Japanese patients had the oldest mean age at diagnosis (74.9 years) and the highest proportion of unmarried individuals and deaths due to cancer or all causes (**Table 1**).

### Comparison of characteristics among Asian American, White, and Black patients with gastric cancer diagnosed between 2000 and 2021

Compared to Asian American patients, both White and Black patients had lower percentages of surgical interventions. White patients had the highest percentage (34%) of gastric cardia cancer. Black patients had the highest percentages of unmarried individuals, residents of the Southern region, and individuals in the lowest income levels. However, they also had the lowest prevalence of grade III/IV cancer (**Table 1**; **Supplementary Table 2**).

### Comparison of cancer-specific and overall survival rates among ethnic subgroups with gastric cancer diagnosed between 2000 and 2021

**Figure 1:**
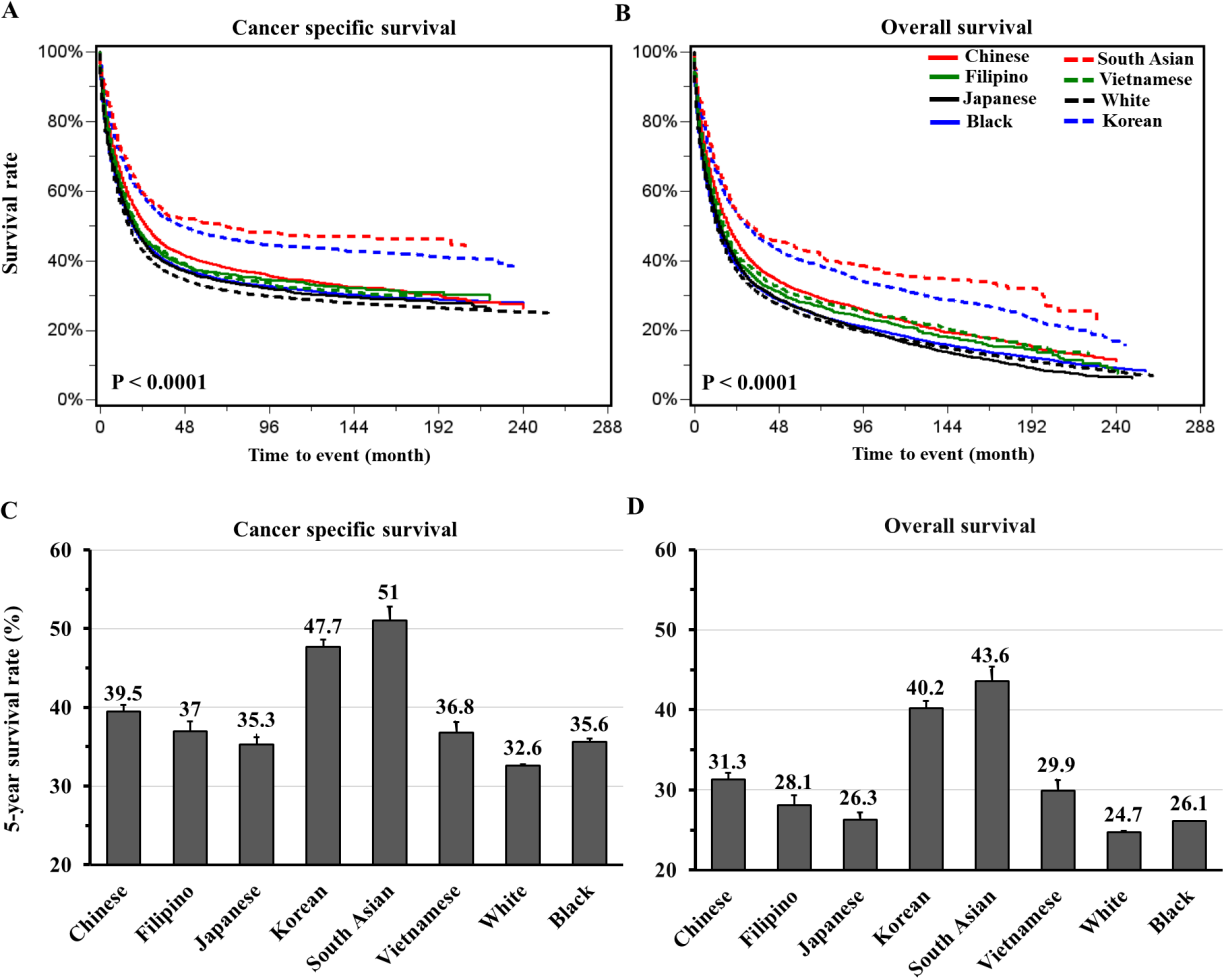
Kaplan–Meier survival curves and five-year rates for patients with gastric cancers diagnosed during 2000–2021. Cancer-specific survival **(A)** and overall survival **(B)** curves. Five-year cancer-specific **(C)** and overall **(D)** survival rates by racial/ethnic subgroup.

Kaplan-Meier survival analyses showed that South Asian patients exhibited the most favorable cancer-specific and overall survival outcomes, followed by Korean patients, both of whom had superior survival outcomes than the other ethnic subgroups (**Figures 1A, B**). The five-year cancer-specific survival rates were 51.0% ± 1.8% for South Asians and 47.7% ± 0.9% for Koreans. Among Asian Americans, Japanese patients had the lowest 5-year cancer-specific survival rate (35.3% ± 0.9%). Black patients had a comparable 5-year cancer-specific survival rate (35.6% ± 0.4%), whereas White patients had an even lower rate (32.6% ± 0.2%) (**Figure 1C**). A similar pattern of disparities in the 5-year overall survival rates was also observed among Asian American, White, and Black patients (**Figure 1D**).

### Univariable and multivariable Cox proportional hazards regression survival analyses of cancer-specific and overall mortality among ethnic subgroups with gastric cancer diagnosed between 2000 and 2021

Univariable Cox proportional hazards regression analyses indicated that South Asian patients displayed a marginally lower risk of cancer-specific mortality than Korean patients (HR = 1.10, 95% CI: 0.99-1.22, P = 0.0730), and a significantly lower overall mortality (HR = 1.11, 95% CI: 1.01-1.21, P = 0.0301). South Asian patients also exhibited significantly reduced risks of cancer-specific and overall mortality compared to all other Asian American subgroups. Among Asian American subgroups, Japanese patients had the highest risk of cancer-specific mortality (HR = 1.60, 95% CI: 1.44-1.77, P<0.0001 vs South Asian) and overall mortality (HR = 1.70, 95% CI: 1.55-1.86, P<0.0001). Black patients exhibited elevated risks of cancer-specific (HR = 1.60, 95% CI: 1.45-1.76, P<0.0001) and overall mortality (HR = 1.68, 95% CI: 1.54-1.83, P<0.0001), whereas White patients experienced slightly higher risks of cancer-specific mortality (HR = 1.70, 95% CI: 1.55-1.87, P<0.0001) and overall mortality (HR = 1.72, 95% CI: 1.59-1.87, P<0.0001) (**Supplementary Table 3**).

In multivariable Cox proportional hazards regression analyses, South Asian patients had risks of cancer-specific mortality (HR = 1.1, 95% CI: 1.0-1.2, P = 0.2984) and overall mortality (HR = 1.0, 95% CI: 0.9-1.1, P = 0.8407) comparable to those of Korean patients. However, South Asian patients exhibited significantly lower risk of cancer-specific mortality than all other Asian American subgroups. They also demonstrated a marginally reduced risk of overall mortality than Chinese patients (HR = 1.1, 95% CI: 1.0-1.2, P = 0.0514), but a significantly reduced risk of overall mortality than Vietnamese (HR = 1.2, 95% CI: 1.0-1.3, P = 0.0062), Filipino (HR = 1.3, 95% CI: 1.2-1.4, P<0.0001), and Japanese patients (HR = 1.2, 95% CI: 1.1-1.3, P<0.0001). White and Black patients exhibited comparable risks of cancer-specific mortality (both HR = 1.4, 95% CI 1.3-1.5, P<0.0001) and overall mortality (both HR = 1.4, 95% CI: 1.3-1.5, P<0.0001) (**Table 2**).

**Table 2:** Multivariable Cox-proportional hazard regression analyses of cancer-specific and overall deaths in patients with gastric cancer diagnosed during 2000–2021.

Variable	Cancer specific death		Overall death	
	HR (95% CI)	P value	HR (95% CI)	P value
Racial/ethnic group				
South Asian	1		1	
Chinese	1.1 (1.0 - 1.2)	0.0276	1.1 (1.0 - 1.2)	0.0514
Korean	1.1 (1.0 - 1.2)	0.2982	1.0 (0.9 - 1.1)	0.8407
Japanese	1.2 (1.1 - 1.4)	<0.0001	1.2 (1.1 - 1.3)	<0.0001
Filipino	1.2 (1.1 - 1.3)	<0.0001	1.3 (1.2 - 1.4)	<0.0001
Vietnamese	1.2 (1.1 - 1.3)	0.0036	1.2 (1.0 - 1.3)	0.0062
White	1.4 (1.3 - 1.5)	<0.0001	1.4 (1.3 - 1.5)	<0.0001
Black	1.4 (1.3 - 1.5)	<0.0001	1.4 (1.3 - 1.5)	<0.0001
Age (year)				
<50	1		1	
50- 59	1.1 (1.0 - 1.1)	<0.0001	1.1 (1.1 - 1.1)	<0.0001
60-69	1.1 (1.1 - 1.2)	<0.0001	1.3 (1.3 - 1.3)	<0.0001
70-79	1.3 (1.3 - 1.4)	<0.0001	1.6 (1.6 - 1.7)	<0.0001
≥80	1.7 (1.7 - 1.8)	<0.0001	2.2 (2.1 - 2.2)	<0.0001
Gender				
Male	1		1	
Female	0.9 (0.9 - 0.9)	<0.0001	0.9 (0.8 - 0.9)	<0.0001
Marital status				
Married	1		1	
Unmarried	1.1 (1.1 - 1.1)	<0.0001	1.2 (1.1 - 1.2)	<0.0001
Unknown	0.9 (0.8 - 0.9)	<0.0001	0.9 (0.9 - 0.9)	<0.0001
Region				
West	1		1	
South	1.1 (1.1 - 1.1)	<0.0001	1.1 (1.1 - 1.1)	<0.0001
Midwest	1.0 (1.0 - 1.1)	0.056	1.0 (1.0 - 1.1)	0.0782
Northeast	0.9 (0.9 - 0.9)	<0.0001	0.9 (0.9 - 0.9)	<0.0001
Income				
<75,000	1		1	
75,000-94,999	1.0 (1.0 - 1.0)	0.0327	1.0 (1.0 - 1.0)	0.0029
≥ 95,000	0.9 (0.9 - 1.0)	<0.0001	0.9 (0.9 - 0.9)	<0.0001
Primary site				
Cardia	1		1	
Not specified	1.1 (1.1 - 1.1)	<0.0001	1.1 (1.1 - 1.1)	<0.0001
Antrum	0.9 (0.9 - 0.9)	<0.0001	0.9 (0.9 - 1.0)	<0.0001
Body	0.9 (0.9 - 0.9)	<0.0001	0.9 (0.9 - 0.9)	<0.0001
Fundus	0.9 (0.9 - 0.9)	<0.0001	0.9 (0.9 - 1.0)	0.0011
Greater	0.9 (0.9 - 0.9)	<0.0001	0.9 (0.9 - 1.0)	<0.0001
Lesser	0.8 (0.8 - 0.9)	<0.0001	0.9 (0.9 - 0.9)	<0.0001
Pylorus	1.0 (0.9 - 1.0)	0.2142	1.0 (1.0 - 1.1)	0.6008
Size (cm)				
≤ 3	1		1	
>3	1.5 (1.4 - 1.5)	<0.0001	1.3 (1.3 - 1.4)	<0.0001
Unknown	1.6 (1.5 - 1.6)	<0.0001	1.5 (1.4 - 1.5)	<0.0001
Stage				
Localized	1		1	
Regional	2.9 (2.8 - 3.0)	<0.0001	2.1 (2.1 - 2.1)	<0.0001
Distant	4.9 (4.8 - 5.0)	<0.0001	3.5 (3.4 - 3.6)	<0.0001
Unknown	1.9 (1.9 - 2.0)	<0.0001	1.5 (1.5 - 1.6)	<0.0001
Histology				
Diffuse	1		1	
Intestinal	0.9 (0.9 - 0.9)	<0.0001	0.9 (0.9 - 0.9)	<0.0001
Other	0.5 (0.5 - 0.5)	<0.0001	0.5 (0.5 - 0.5)	<0.0001
Grade				
I	1		1	
II	1.9 (1.8 - 2.0)	<0.0001	1.5 (1.4 - 1.6)	<0.0001
III/IV	2.6 (2.5 - 2.7)	<0.0001	1.9 (1.9 - 2.0)	<0.0001
Unknown	1.9 (1.8 - 2.0)	<0.0001	1.5 (1.5 - 1.6)	<0.0001
Surgery				
No	1		1	
Yes	0.4 (0.4 - 0.4)	<0.0001	0.4 (0.4 - 0.4)	<0.0001
Chemotherapy				
No	1		1	
Yes	0.5 (0.5 - 0.5)	<0.0001	0.5 (0.5 - 0.5)	<0.0001
Radiotherapy				
No	1		1	
Yes	1.0 (1.0 - 1.0)	0.0897	0.97 (0.95 – 0.98)	<0.0001

CI, confidence interval; HR, Hazard ratio.

Our data also identified that older ages, males, unmarried individuals, residents of the Southern region (vs the West region), cancer at the gastric cardia, larger tumor size, a more advanced stage, diffuse histological type, higher grade, and those who did not receive surgical intervention or chemotherapy were associated with increased risks of both cancer-specific and overall mortality. In contrast, patients who resided in the Northeast region (vs the West region) were associated with more favorable cancer-specific and overall mortality. Radiation therapy was only significantly correlated with a lower risk of overall mortality but did not show a significant association with cancer-specific mortality (**Table 2**).

### Comparison of cancer-specific and overall survival outcomes among Asian American patients with gastric cancer after stratification by subgroups

Kaplan-Meier survival curves showed that South Asian patients had the most favorable cancer-specific and overall survival outcomes among Asian American subgroups across multiple subgroups, including patients aged < 70 years (**Supplementary Figures 1A, B**), those with non-cardia gastric cancer (**Supplementary Figures 1C, D**), non-diffuse histology (**Supplementary Figures 1E, F**), those who did not undergo surgery (**Supplementary Figures 1G, H**), those who did not receive chemotherapy (**Supplementary Figures 2A, B**) or radiotherapy (**Supplementary Figures 2C, D**), and patients with distant-stage disease (**Supplementary Figures 2E, F**). Additionally, South Asian patients had the most favorable overall survival among Asian American patients with localized-stage disease (**Supplementary Figure 2G**) and among those who underwent surgery (**Supplementary Figure 2H**).

South Asians patients demonstrated cancer-specific and overall survival outcomes comparable to those of Korean patients, but superior to those of the other Asian American subgroups among both male (**Supplementary Figures 3A, B**) and female patients (**Supplementary Figures 3C, D**). Similarly, cancer-specific survival was comparable among these subgroups in patients with localized-stage disease (**Supplementary Figure 3E**) and among those who underwent surgery (**Supplementary Figure 3F**).

In contrast, Korean patients exhibited the most favorable cancer-specific and overall survival outcomes among Asian American subgroups in patients aged ≥ 70 years (**Supplementary Figures 4A, B**), those with diffuse histology (**Supplementary Figures 4C, D**), and those who received radiotherapy (**Supplementary Figures 4E, F**).

However, South Asian, Korean, and one or more other Asian American subgroups exhibited comparable cancer-specific survival and overall survival outcome in patients with regional-stage tumors (**Supplementary Figures 5A, B**), patients with gastric cardia tumors (**Supplementary Figures 5C, D**), and those who received chemotherapy (**Supplementary Figures 5E, F**).

**Figure 2:**
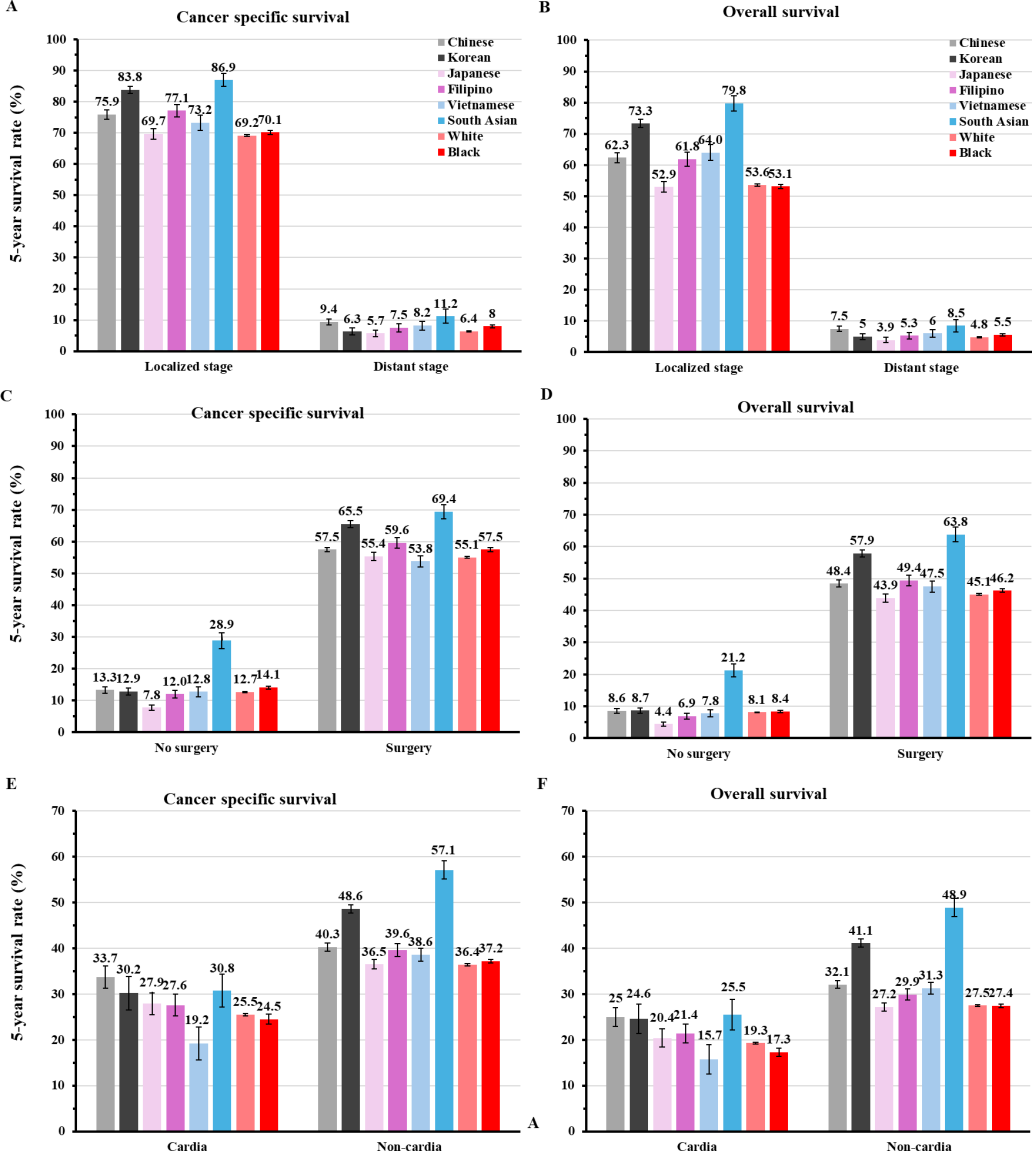
Five-year cancer-specific and overall survival rates among patients with gastric cancers by racial/ethnic subgroup after subgroup stratification. Five-year cancer-specific survival **(A)** and overall survival **(B)** rates are shown for patients with localized-stage and distant-stage gastric cancer. Five-year cancer-specific survival **(C)** and overall survival **(D)** rates are shown for patients with surgery or without surgery. Five-year cancer-specific survival **(E)** and overall survival **(F)** rates are shown for patients with cardia or non-cardia gastric cancer.

Five-year cancer-specific and overall survival rates among Asian American patients stratified by disease stage (**Figures 2A, B**), surgical status (**Figures 2C, D**), and tumor site (**Figures 2E, F**), whereas corresponding results for all stratified subgroups are presented in **Supplementary Table 4**. In addition, univariable Cox proportional hazards regression analyses revealed these patterns of disparities in the risks of cancer-specific and overall mortality among Asian American ethnic subgroups across the stratified subgroups (**Supplementary Table 4**).

### Comparison of cancer-specific and overall survival in gastric cancer between 2000–2010 and 2011–2021

**Figure 3:**
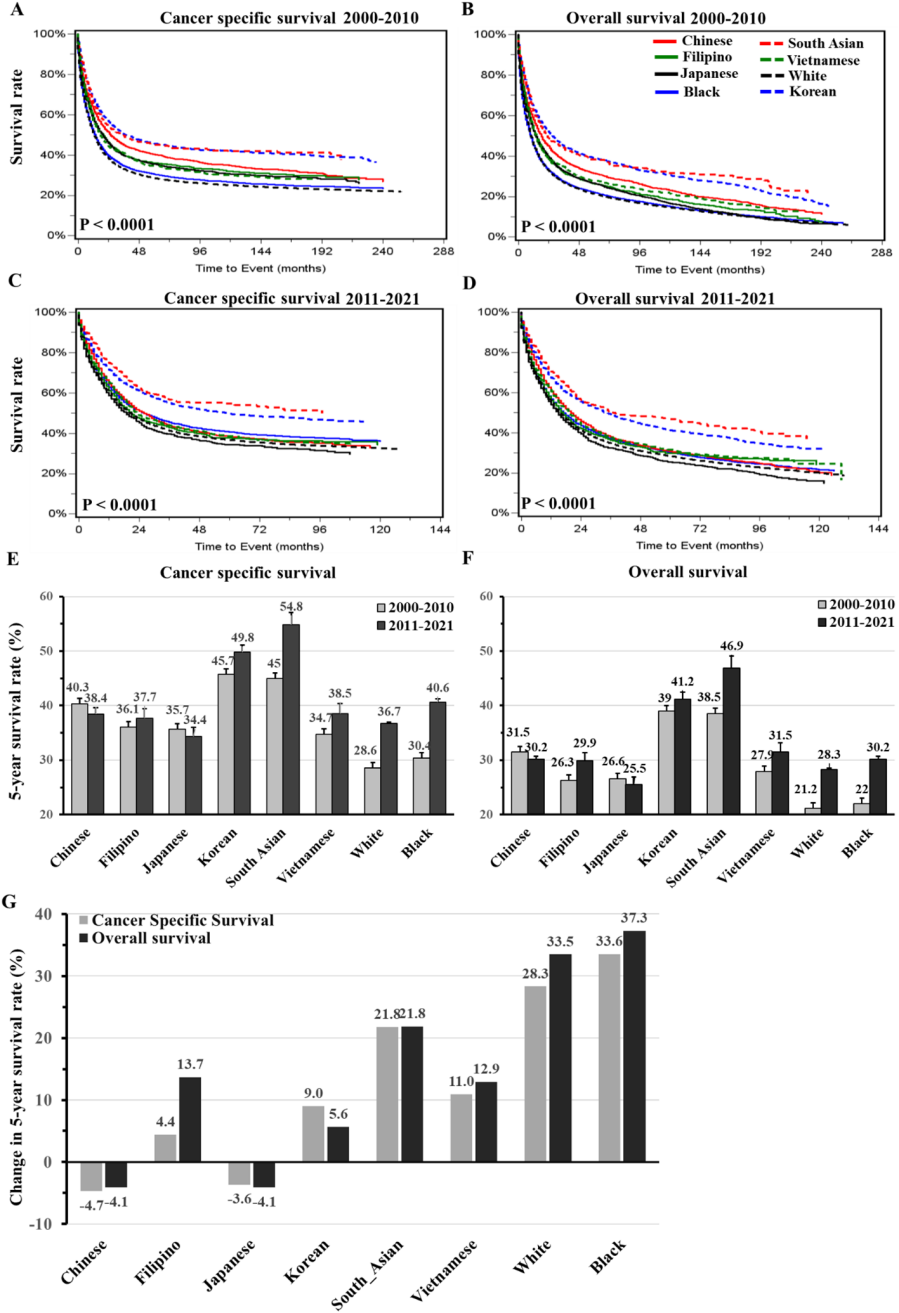
Kaplan–Meier cancer-specific survival and overall survival curves and corresponding 5-year survival rates among patients with gastric cancers diagnosed during 2000–2010 and 2011–2021. Cancer specific survival and overall survival curves for patients diagnosed during 2000–2010 [**(A, B)**, respectively] and between 2011–2021 [**(C, D)**, respectively). Five-year cancer-specific survival **(E)** and overall survival **(F)** rates for patients diagnosed during two periods. Changes in 5-year cancer-specific and overall survival rate between the two diagnostic periods **(G)**.

During the 2000–2010 period, Korean and South Asian patients had comparable 5-year cancer-specific survival rates (45.7% ± 1.2% vs 45.0% ± 2.9%, respectively) (**Figure 3A, E**) and overall survival rates (39.0% ± 1.1% vs 38.5% ± 2.7%, respectively) (**Figures 3B, F**), both of which were higher than those observed in the other Asian American subgroups. White and Black patients exhibited the lowest 5-year cancer-specific and overall survival rates during this period. During the 2011–2021 period, South Asians achieved the highest 5-year cancer-specific rate of 54.8% ± 2.3% (**Figures 3C, E**) and overall survival rate of 46.9% ± 2.2% (**Figures 3D, F**), whereas Japanese had the worst 5-year cancer-specific (34.4% ± 1.6%) (**Figures 3C, E**) and overall survival rates (25.5% ± 1.4%) (**Figures 3D, F**). When comparing the two time periods, significant improvements in cancer-specific survival were observed among Korean, Vietnamese, South Asian, White, and Black patients, whereas no significant improvements were observed among Chinese, Japanese, or Filipino patients (**Supplementary Figures 6A–H**). For overall survival, significant improvements were observed among Filipino, Vietnamese, South Asian, White, and Black patients, whereas Chinese, Korean, and Japanese patients showed no significant changes over time (**Supplementary Figures 7A–H**). Among Asian American subgroups, South Asian patients experienced the greatest improvement in both 5-year cancer-specific survival and overall survival, with increases of 21.8% for each outcome between the two periods. However, White and Black patients demonstrated even larger relative improvements in both 5-year cancer-specific and overall survival rates (**Figure 3G**).

Univariable Cox proportional hazards regression survival analyses indicated that, during 2000-2010, South Asian patients had risks of cancer-specific and overall mortality comparable to those of Korean patients, but lower than those of the other American subgroups. Multivariable analysis revealed no significant difference in the risks of cancer-specific or overall mortality among Asian American subgroups during this period (**Table 3**). During 2011-2021, univariable Cox proportional hazards survival analyses similarly showed that South Asian patients had risks of cancer-specific and overall mortality comparable to those of Korean patients, but lower than those of the other American subgroups. In multivariable analyses, South Asian patients continued to have a risk of cancer-specific mortality comparable to that of Korean patients (HR = 1.0, 95% CI: 0.9-1.2, P = 0.6456), but significantly lower than that of the other Asian American subgroups. For overall mortality, South Asian patients had risks comparable to those of Korean (HR = 1.0, 95% CI: 0.9-1.1, P = 0.7239) and Chinese patients (HR = 1.1, 95% CI: 1.0-1.2, P = 0.1051), but significantly lower than those of the remaining Asian American subgroups (**Table 3**).

**Table 3:** Univariable and multivariable Cox proportional hazards regression analyses of cancer-specific and overall death by racial/ethnic group among patients with gastric cancer in periods of 2000–2010 and 2011–2021.

Variable	Cancer-specific death				Overall death			
	Univariable		Multivariable*		Univariable		Multivariable	
Period	HR (95% CI)	P value	HR (95% CI)	P value	HR (95% CI)	P value	HR (95% CI)	P value
2000–2010								
South Asian	1		1		1		1	
Chinese	1.2 (1.0 - 1.4)	0.0364	1.0 (0.8 - 1.1)	0.6426	1.2 (1.1 - 1.4)	0.003	1.0 (0.8 - 1.1)	0.6342
Filipino	1.3 (1.1 - 1.5)	0.0016	1.1 (0.9 - 1.3)	0.3791	1.4 (1.2 - 1.6)	<0.0001	1.1 (1.0 - 1.3)	0.0753
Japanese	1.3 (1.2 - 1.6)	0.0002	1.0 (0.9 - 1.2)	0.7226	1.5 (1.3 - 1.7)	<0.0001	1.1 (0.9 - 1.2)	0.4901
Korean	1.0 (0.8 - 1.2)	0.8199	1.0 (0.8 - 1.1)	0.7724	1.0 (0.9 - 1.2)	0.9838	0.9 (0.8 - 1.1)	0.4204
Vietnamese	1.4 (1.1 - 1.6)	0.0006	1.1 (0.9 - 1.3)	0.2501	1.3 (1.2 - 1.6)	0.0002	1.1 (0.9 - 1.3)	0.3170
White	1.6 (1.4 - 1.9)	<0.0001	1.3 (1.1 - 1.5)	0.0009	1.7 (1.5 - 1.9)	<0.0001	1.3 (1.1 - 1.5)	0.0002
Black	1.6 (1.4 - 1.8)	<0.0001	1.4 (1.2 - 1.6)	<.0001	1.7 (1.5 - 1.9)	<0.0001	1.4 (1.2 - 1.6)	<0.0001
2011–2021								
South Asian	1		1		1		1	
Chinese	1.5 (1.3 - 1.7)	<0.0001	1.2 (1.0 - 1.3)	0.0412	1.5 (1.3 - 1.6)	<.0001	1.1 (1.0 - 1.2)	0.1051
Filipino	1.6 (1.4 - 1.9)	<0.0001	1.4 (1.2 - 1.6)	<0.0001	1.5 (1.4 - 1.8)	<.0001	1.3 (1.2 - 1.5)	<0.0001
Japanese	1.8 (1.5 - 2.0)	<0.0001	1.2 (1.0 - 1.4)	0.0115	1.8 (1.5 - 2.0)	<.0001	1.1 (1.0 - 1.3)	0.0343
Korean	1.1 (1.0 - 1.3)	0.0751	1.0 (0.9 - 1.2)	0.6456	1.1 (1.0 - 1.3)	0.0735	1.0 (0.9 - 1.1)	0.7239
Vietnamese	1.5 (1.3 - 1.8)	<0.0001	1.2 (1.0 - 1.4)	0.0137	1.5 (1.3 - 1.7)	<0.0001	1.2 (1.0 - 1.3)	0.0268
White	1.7 (1.5 - 1.9)	<0.0001	1.3 (1.2 - 1.5)	<0.0001	1.6 (1.5 - 1.8)	<0.0001	1.3 (1.2 - 1.5)	<0.0001
Black	1.5 (1.3 - 1.7)	<0.0001	1.3 (1.2 - 1.5)	<0.0001	1.6 (1.4 - 1.8)	<0.0001	1.3 (1.2 - 1.5)	<0.0001

*Multivariable survival analyses were adjusted for age, marital status, region, anatomical site, size stage, histological type, grade, surgery, chemotherapy, and radiotherapy.

### Comparison of patient characteristics in gastric cancer between 2000–2010 and 2011–2021

**Figure 4:**
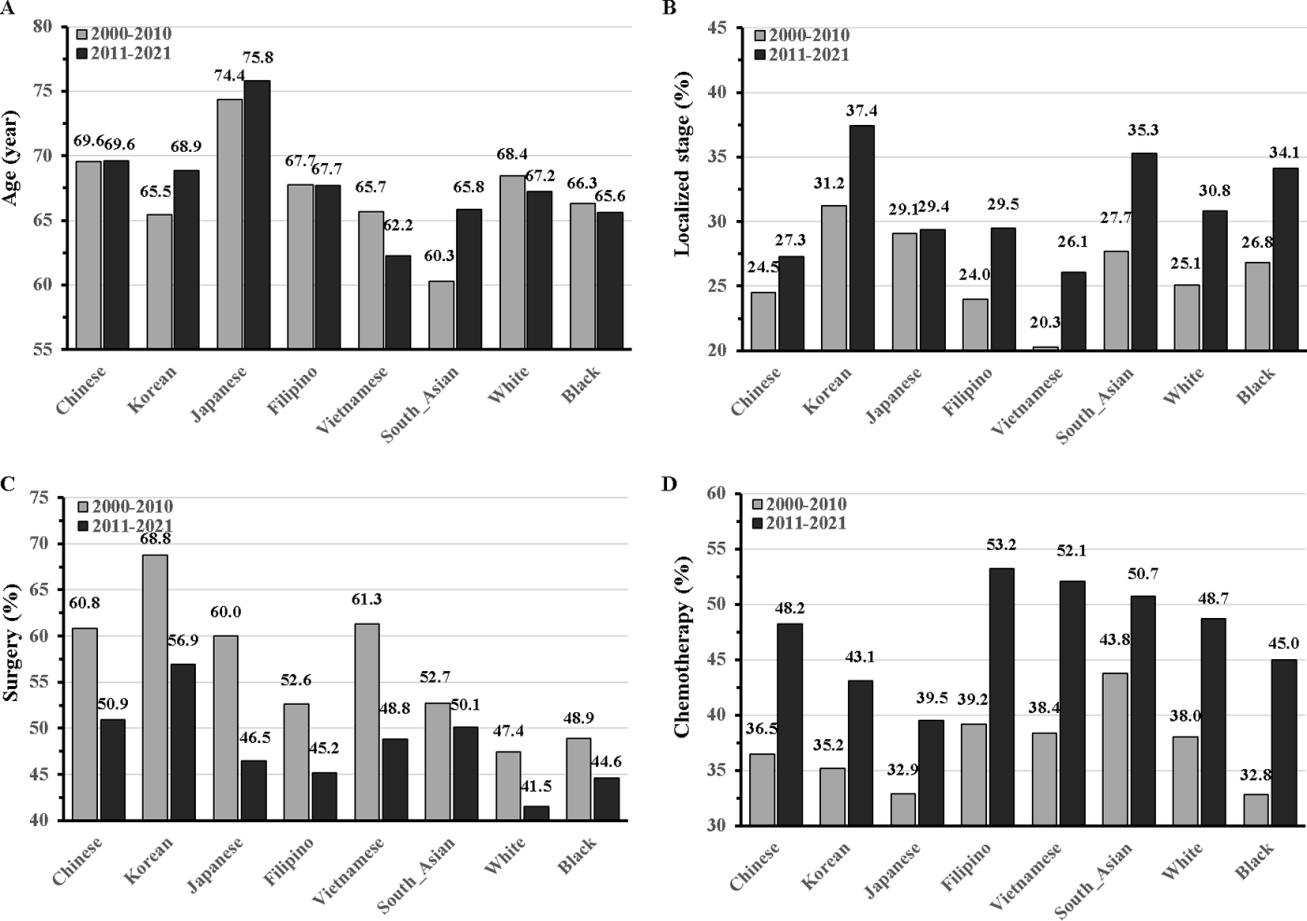
Demographic and clinical features in patients with gastric cancers diagnosed during 2000–2010 and 2011-2021. Comparison of mean age **(A)**, localized-stage disease **(B)**, receipt of surgery **(C)**, and receipt of chemotherapy **(D)** between two diagnostic periods across racial/ethnic subgroup.

Detailed features of patients with gastric cancer for the periods of 2000–2010 and 2011–2021 are presented in **Supplementary Table 5** and **6**, respectively. Our data revealed that Vietnamese, White, and Black patients had a decrease in mean age at diagnosis, while patients of other ethnic subgroups experienced an increase in mean age between the two periods (**Figure 4A**). The proportion of localized -stage gastric cancer increased in all ethnic subgroups except Japanese patients (**Figure 4B**). Meanwhile, the proportions of patients undergoing surgical interventions declined (**Figure 4C**), whereas the use of chemotherapy increased in all ethnic subgroups from the first decade to the second (**Figure 4D**). In addition, all ethnic subgroups exhibited increased proportions of cardia gastric cancer, accompanied by decreased proportions of grade III/IV tumor, diffusive histological type, and receipt of radiotherapy (**Supplementary Figures 8A–D**).

## Discussion

Using the SEER database (2000–2021), our study determined clinicopathological characteristics and survival disparities in Asian American, White, and Black patients with gastric cancer. We observed substantial heterogeneity within Asian American ethnic subgroups. Notably, Asian American patients exhibited a new pattern of survival disparity, with South Asian patients displaying the highest 5-year cancer-specific and overall survival rates across the overall cohort and major stratified subgroups. After adjusting for relevant prognostic factors, South Asian and Korean patients showed the most favorable, whereas White and Black patients still had the poorest survival outcomes. Temporal analyses revealed that Korean, Filipino, Vietnamese, and South Asian patients showed improvements in survival outcomes from 2000–2010 to 2011-2021, with South Asian patients showing the greatest improvements, whereas Chinese and Japanese patients did not. However, all ethnic subgroups exhibited changes in the same direction in most clinicopathological features between the two periods, including increased proportions of early-stage disease, cardia gastric cancer, and chemotherapy use, along with decreased use of surgery and radiotherapy.

Our current study observed a new pattern of survival disparity among Asian Americans with gastric cancer, wherein South Asian patients exhibited the most favorable 5-year cancer-specific and overall survival rates, while Japanese patients demonstrated the lowest rates. The more favorable survival in South Asian and Korean patients can be partially correlated with their more advantageous demographic and clinicopathological characteristics. South Asian patients were diagnosed at a younger average age and had higher proportions of being married, presenting with grade I tumors, and receiving chemotherapy. All these factors are generally associated with improved survival outcomes. Similarly, Korean patients showed higher proportions of localized-stage cancer and surgical intervention, along with the least proportion of tumors located at the cardia, a site linked to poorer prognosis. It is worth noting that South Asians had higher proportions of males and tumors located at the cardia, both of which are generally associated with worse survival outcomes. These findings suggest that the superior survival observed among South Asian patients cannot be fully explained by a uniformly favorable clinicopathologic profile. Rather, their survival advantage likely reflects a complex interplay of both favorable and unfavorable prognostic factors, as well as other potential influences beyond the measured baseline characteristics. After adjusting for other prognostic factors, multivariable analysis indicated that South Asian patients had cancer-specific and overall survival outcomes comparable to Korean patients, but more favorable compared to other Asian American ethnic subgroups. This indicates that additional unmeasured factors, such as genetic predispositions, lifestyle, environmental exposures, or access to culturally appropriate healthcare, may also attribute to their more favorable survival outcomes (17).

In contrast to our finding, an earlier study by Jin et al. (11) using the older SEER database (2000-2012) reported that Koreans exhibited the highest age-standardized 5-year cancer-specific survival rate. No significant differences in cancer-specific survival were observed among the same six Asian American ethnic subgroups after controlling for prognostic factors (11). Likewise, Huang et al. (13), using an earlier SEER cohort (1990–2014), reported similar 5-year overall survival rates between Korean and South Asian patients with non-cardia gastric cancer (13). In contrast, our study found that South Asian patients had substantially higher cancer-specific and overall survival rates than Korean and other Asian American subgroups, particularly among patients with non-cardia gastric cancer. Additional studies have also identified Korean patients as having the most favorable survival outcomes. For example, analyses of the National Cancer Data Base involving patients of Korean, Japanese, and Filipino ancestry with gastric or esophageal adenocarcinoma diagnosed between 2004 and 2013 found that Koreans had the most favorable survival outcomes, whereas Filipinos had the poorest outcomes (13). Similarly, a study of Asian American patients with gastric adenocarcinoma treated between 1988 and 2006 using data from the Los Angeles County Cancer Surveillance Program reported superior survival among Korean patients (17). Importantly, these earlier studies were based on cohorts diagnosed one or two decades ago. The discrepancies between prior reports and our current findings may largely reflect evolving and heterogeneous survival patterns among Asian American ethnic subgroups over time. Our temporal analyses demonstrated substantial improvements in survival among South Asian patients between 2000–2010 and 2011–2021, whereas survival gains were more modest or absent in several other Asian American subgroups. These differing temporal trends may have altered the relative survival rankings of Asian American subgroups observed in our current study.

The differing survival outcomes observed among Asian American patients with gastric cancer in the earlier decade and the divergent changes in survival over the subsequent two decades have led to the emergence of a new survival pattern across Asian American ethnic subgroups. This pattern appears to appears to stem from a combination of different baseline survival rates and varying rates of survival improvement over time. Between 2000–2010 and 2011-2021, Chinese and Japanese patients displayed no significant change in survival, whereas other Asian American subgroups demonstrated varying degrees of survival gains, with South Asian patients showing the greatest increases in both cancer-specific and overall survival rates. Consequently, South Asian patients achieved the highest cancer-specific and overall survival rates, whereas Japanese patients had the poorest survival outcomes in the most recent decade. The improved cancer-specific and overall survival among Asian American patients as well as among White and Black patients likely reflect better therapy regimens and enhanced access to care over recent decades (18–20).

However, most Asian American ethnic subgroups exhibited changes in in many clinicopathological features at the same direction during the past two decades. All ethnic subgroups had increased proportions of localized-stage disease, cardia gastric cancer, and chemotherapy use, along with decreased proportions of diffusive-histology, grade III/IV tumors, surgical intervention and radiotherapy. The changes in treatment pattern are consistent with greater reliance on systemic therapy (chemotherapy) and reduced use of surgery in recent years (21). Nevertheless, despite these broadly consistent changes in clinicopathological features, survival trends differed substantially across Asian American subgroups. This discordance suggests that factors beyond the measured clinicopathologic variables may contribute to the observed differences in survival trajectories. Identifying these factors could provide important insights for developing more effective prevention strategies and tailored therapeutic approaches to improve outcomes among diverse Asian American populations.

Several factors may help explain the greatest improvement in survival among South Asian patients with gastric cancer over the study periods. South Asians had the fast-growing subgroup among the six major Asian American subgroups and had a higher proportion of recent immigrants, whereas Japanese Americans tend to have the fewest new arrivals (22). Recent immigrants are more likely to retain cultural dietary habits and lifestyle behaviors. Traditional South Asian diets, rich in phytochemicals and plant-based nutrients, have been associated with cancer-protective effects (23, 24). In addition, recent immigrants may benefit from targeted public health initiatives and screening programs, potentially facilitating earlier detection and improved outcomes. Consistent with this, foreign-born Asian American patients with gastric cancer have been reported to have lower mortality risk than their US-born counterparts (10). Additionally, recent immigrants, particularly South Asians, often have higher educational attainment, higher median household income, and higher overall socioeconomic status compared with other Asian immigrant groups (25). Higher socioeconomic status is associated with improved access to preventative care, higher quality of health care, greater awareness of cancer, and increased uptake of screening, diagnosis and treatment (26). Taken together, immigration patterns, cultural practices, public health engagement, and socioeconomic advantage, may contribute to the observed best improvement in survival outcomes among South Asian patients with gastric cancer.

Encouragingly, our study observed that the proportion of localized-stage gastric cancer increased from 2000–2010 to 2011-2021. However, an average of 29% of Asian American patients was diagnosed with localized-stage gastric cancer. The low proportion of localized-stage disease has been previously reported (11, 13) and is strongly correlated with their poor 5-year survival rates. Because early-stage gastric cancer is often asymptomatic, effective screening strategies are essential to enable detection before the onset of clinical symptoms (27). Population-based gastric cancer screening has been associated with increased early diagnosis and improved survival in East Asian countries such as Japan and Korea (27–29). Prior studies have also demonstrated that a one-time esophagogastroduodenoscopy (EGD) is a cost-effective screening approach when properly targeted to high-risk populations (30–32). However, no national screening guideline for gastric cancer screening currently exists in the US. Given the significantly higher incidence of gastric cancer among Asian Americans, it is essential to develop tailored screening strategies to improve early detection and survival outcomes (33, 34).

Our data revealed that there are substantial ethnic variations in proportion of cardia and non-cardia gastric cancer among patients with gastric cancer. White patients displayed much higher proportions of cardia gastric cancer than other ethnic subgroups. Consistently, a previous study using the California Cancer Registry data (2011-2015) reported that non-Hispanic White patients had the highest incidence of cardia gastric cancer (6). Cardia and non-cardia gastric cancer are associated with distinct risk factors. *Helicobacter pylori* infection is primarily linked to non-cardia gastric cancer, whereas obesity and gastroesophageal reflux are more strongly associated with cardia gastric cancer (35, 36). Our study also showed that the proportion of cardia gastric cancer increased in all ethnic subgroups over the past two decades, likely reflecting the increasing prevalence of obesity and gastroesophageal reflux (37). Cardia gastric cancer is associated with worse 5-year survival compared to non-cardia gastric cancer (6, 33). Taken together, these findings highlight the importance of exploring the underlying risk factors that shape ethnic disparities in gastric cancer tumor location and underscore the need for targeted prevention and risk-reduction strategies.

Previous studies reported that White patients experienced worse 5-year cancer-specific and overall survival rates compared to any of the six major Asian American ethnic subgroups (8–12). Consistent with these findings, our study showed that both White and Black patients had worse 5-year survival rates than Asian American patients during 2000-2010. Due to differential improvement in survival from 2000–2010 to 2011-2021, both White and Black patients had better 5-year survival rates than Japanese patients, and Black patients had better 5-year survival rates than White patients in the most recent decade. In line with our results, a recent study showed that Black patients had better median overall survival rates than White patients among those who underwent surgery (12). The relatively better survival in Black patients may be partially correlated with their younger mean ages at diagnosis, lower proportions of males, tumors at the cardia, grade III/IV tumors, higher proportions of tumors at the localized stage, and a greater rate of surgical intervention. After adjusting for prognostic factors, multivariable survival analyses indicated that White and Black patients had comparable risks of mortality, but both groups continued to have significantly worse survival outcomes than Asian American ethnic subgroups. These persistent disparities likely reflect complex interactions among genetic, cultural, environmental, and healthcare-related factors, which warrant further investigation (10, 38).

Several limitations in this retrospective study should be acknowledged. Because the reference population data required for standardization are not available in the SEER database, we are unable to calculate age-standardized incidence and mortality rates. Consequently, we could not directly compare the population-level burden of gastric cancer across these ethnic subgroups. The SEER database does not capture birthplaces of Asian Americans, precluding differentiation between immigrant and US-born Asian American populations. Patients in the SEER database are primarily from the Northeast and West regions, potentially limiting generalizability to other regions. Although the household income is available, other key socioeconomic variables, such as insurance status, education level, and occupation, are not included. The database lacks important lifestyle and risk factors, such as smoking status, family history, diet, and physical activity. Additionally, clinical details are unavailable, such as comorbidities, surgical margins, and postoperative complications. Furthermore, data on disease recurrence and subsequent treatments during follow-up are not recorded, despite their potential influence on survival outcomes. All these limitations hinder our ability to elucidate the mechanisms accounting for the observed disparities. Nonetheless, our study provides valuable and updated evidence highlighting the substantial heterogeneity in clinicopathological characteristics, survival, and their temporal evolution among Asian American subgroups.

## Conclusions

Our findings highlight substantial heterogeneity in clinicopathological features, survival, and their dynamic evolution over time among Asian American patients with gastric cancer. South Asian patients demonstrated the greatest improvement and emerged as the subgroup with the most favorable survival outcomes. The proportions of cardia gastric cancer and use of systemic therapy increased in the most recent decade. Further research is needed to elucidate the factors driving these trends. Targeted efforts are warranted to develop effective prevention strategies to reduce incidence, implement cost-effective screening to enhance early detection, and mitigate survival disparities among Asian American populations.

## Data Availability

Publicly available datasets were analyzed in this study. This data can be found here: https://seer.cancer.gov/data/access.html#researchonly.
