# Supplementary material for: Revisiting gastric cancer disparities in Asian American subgroups: insights from the SEER database
Source: Front Oncol. 2026 Jun 16;16:1858617. doi: 10.3389/fonc.2026.1858617 (PMC13314428; doi:10.3389/fonc.2026.1858617)

Supplementary data

Table S1. Distribution of patients with gastric cancer with known survival time by race/ethnicity diagnosed during 2000-2021

Race / ethnicity	Frequency	Percent
**Included (n=131,793)**		
**White**	97,407	71.03
**Black**	18,065	13.17
**Asian Americans (n=16,321)**		
**Chinese**	4,340	3.16
**Korean (1988+*)**	3,787	2.76
**Japanese**	3,207	2.34
**Filipino**	2,173	1.58
**Vietnamese (1988+)**	1,775	1.29
**South Asian (n= 1,039)**		
**Asian Indian (2010+)**	669	0.49
**Asian Indian or Pakistani, NOS (1988+)**	321	0.23
**Pakistani (2010+)**	49	0.04
**Excluded (n=5,339)**		
**Hawaiian**	659	0.48
**Laotian (1988+)**	141	0.1
**Thai (1994+)**	105	0.08
**Samoan (1991+)**	223	0.16
**Kampuchean (1988+)**	136	0.1
**Pacific Islander, NOS (1991+)**	115	0.08
**Hmong (1988+)**	86	0.06
**Tongan (1991+)**	71	0.05

**Micronesian, NOS (1991+)**	42	0.03
**Fiji Islander (1991+)**	32	0.02
**Guamanian, NOS (1991+)**	25	0.02
**Chamorran (1991+)**	7	0.01
**Polynesian, NOS (1991+)**	7	0.01
**Melanesian, NOS (1991+)**	2	0
**New Guinean (1991+)**	1	0
**Tahitian (1991+)**	1	0
**Other Asian (1991+)**	1,562	1.14
**American Indian/Alaska Native**	1,293	0.94
**Other**	423	0.31
**Unknown**	408	0.3

* The year indicates when that ethnicity began to be recorded as a distinct ethnic category in the SEER database for newly diagnosed patients.

Table S2. Characteristics of Asian American (overall), white, and black patients with gastric cancer diagnosed during 2000-2021

Variable	Overall<br>n (%)	White<br>n (%)	Black<br>n (%)	P value
	16,321 (12)	97,407 (74)	18,065 (14)	
**Age (year)**				
**Mean ± SD**	68.9 ± 13.9[a]	67.8 ± 14.0[b]	65.9 ± 13.8[c]	P<0.0001
**Median**	71	69	67	
**Group**				P<0.0001
**<50**	1,651 (10)	10,537 (11)	2,250 (12)	
**50- 59**	2,277 (14)	15,385 (16)	3,443 (19)	
**60-69**	3,682 (23)	23,471 (24)	4,729 (26)	
**70-79**	4,604 (28)	26,015 (27)	4,406 (24)	
**≥80**	4,107 (25)	21,999 (23)	3,237 (18)	
**Gender**				P<0.0001
**Male**	9,221 (57)	60267 (62)	10,179 (56)	
**Female**	7,100 (44)	37,140 (38)	7,886 (44)	
**Marital status**				P<0.0001
**Married**	10,672 (65)	55,245 (57)	6,899 (38)	
**Unmarried**	4,931 (30)	36,915 (38)	9,976 (55)	
**Unknown**	718 (4)	5,247 (5)	1,190 (7)	
**Region**				P<0.0001
**West**	14,200 (87)	55,449 (57)	5,204 (29)	
**South**	651 (4)	18,058 (19)	9,153 (51)	
**Midwest**	53 (0)	3,835 (4)	120 (1)	
**Northeast**	1,417 (9)	20,065 (21)	3,588 (20)	

**Income ($)**				P<0.0001
**<70,000**	1,305 (8)	28,137 (29)	7,892 (44)	
**70,000-84,999**	6,032 (37)	36,816 (38)	6,202 (34)	
**≥ 85,000**	8,984 (55)	32,454 (33)	3,971 (22)	
**Primary site**				P<0.0001
**Antrum**	4,489 (28)	13,701 (14)	4,024 (22)	
**Body**	1,950 (12)	9,854 (10)	2,094 (12)	
**Cardia**	2,054 (13)	32,766 (34)	2,297 (13)	
**Fundus**	665 (4)	4,864 (5)	1,060 (6)	
**Greater curvature**	821 (5)	3,900 (4)	967 (5)	
**Lesser curvature**	1,888 (12)	6,155 (6)	1,602 (9)	
**Pylorus**	513 (3)	2,137 (2)	644 (4)	
**Overlapping or not specified**	3,941 (24)	24,030 (25)	5,377 (30)	
**Size (cm)**				P<0.0001
**≤ 3**	3,353 (21)	24,069 (18)	2,943 (16)	
**>3**	6,418 (39)	45,402 (34)	6,840 (38)	
**Unknown**	6,550 (40)	62,322 (47)	8,282 (46)	
**Stage**				P<0.0001
**Localized**	4,709 (29)	27,368 (28)	5,544 (31)	
**Regional**	4,915 (30)	24,334 (25)	4,373 (24)	
**Distant**	5,048 (31)	35,016 (36)	6,119 (34)	
**Unknown**	1,649 (10)	10,689 (11)	2,029 (11)	
**Histology**				P<0.0001
**Diffuse**	3,994 (24)	19,189 (20)	2,948 (16)	
**Intestinal**	9,570 (59)	57,048 (59)	10,171 (56)	

**Other**	2,757 (17)	21,170 (22)	4,946 (27)	
**Grade**				P<0.0001
**I**	961 (6)	7,879 (8)	1,739 (10)	
**II**	3,271 (20)	19,548 (20)	3,701 (20)	
**III/IV**	9121 (56)	4,7492 (49)	7,952 (44)	
**Unknown**	2,968 (18)	22,488 (23)	4,673 (26)	
**Surgery**				P<0.0001
**No**	7,239 (44)	54,274 (56)	9,652 (53)	
**Yes**	9,082 (56)	43,133 (44)	8,413 (47)	
**Chemotherapy**				P<0.0001
**No**	9,497 (58)	54,865 (56)	10,956 (61)	
**Yes**	6,824 (42)	42,542 (44)	7,109 (39)	
**Radiotherapy**				P<0.0001
**No**	13,179 (81)	76,041 (78)	15,167 (84)	
**Yes**	3,142 (19)	21,366 (22)	2,898 (16)	
**Cancer specific death**				P<0.0001
**No**	7,487 (46)	38,709 (40)	7,785 (43)	
**Yes**	8,834 (54)	58,698 (60)	10,280 (57)	
**Overall death**				P<0.0001
**No**	4,828 (30)	23,083 (24)	4,509 (25)	
**Yes**	11,493 (70)	74,324 (76)	13,556 (75)	

Table S3. Univariable Cox proportional hazards regression analyses of cancer specific and overall survival in patients with gastric cancer by racial/ethnic group.

Variable	Cancer-specific survival		Overall survival	
**Races**	**HR (95% CI)**	**P value**	**HR (95% CI)**	**P value**
**South Asian**	1		1	
**Chinese**	1.39 (1.25 - 1.54)	<0.0001	1.39 (1.27 - 1.53)	<0.0001
**Filipino**	1.50 (1.34 - 1.67)	<0.0001	1.54 (1.40 - 1.70)	<0.0001
**Japanese**	1.60 (1.44 - 1.77)	<0.0001	1.70 (1.55 - 1.86)	<0.0001
**Korean**	1.10 (0.99 - 1.22)	0.0730	1.11 (1.01 - 1.21)	0.0301
**Vietnamese**	1.49 (1.33 - 1.67)	<0.0001	1.45 (1.31 - 1.60)	<0.0001
**White**	1.70 (1.55 - 1.87)	<0.0001	1.72 (1.59 - 1.87)	<0.0001
**Black**	1.60 (1.45 - 1.76)	<0.0001	1.68 (1.54 - 1.83)	<0.0001

Table S4. Five-year cancer-specific and overall survival rates and Cox proportional hazards regression analyses among gastric cancer patients by racial/ethnic group after subgroup stratification.

Variable	Cancer-specific survival			Overall survival		
	5-year survival rate (%)	Univariable Cox regression		5-year survival rate (%)	Univariable Cox regression	
Subgroup		HR (95% CI)	P value		HR (95% CI)	P value
< 70 years						
South Asian	56.0 ± 2.1	1		50.7 ± 2.1	1	
Chinese	45.0 ± 1.3	1.39 (1.22 - 1.59)	<0.0001	40.0 ± 1.2	1.32 (1.17 - 1.49)	<0.0001
Filipino	36.9 ± 1.6	1.71 (1.48 - 1.97)	<0.0001	32.2 ± 1.5	1.64 (1.44 - 1.87)	<0.0001
Japanese	43.6 ± 1.8	1.45 (1.25 - 1.68)	<0.0001	37.5 ± 1.7	1.46 (1.27 - 1.67)	<0.0001
Korean	49.1 ± 1.2	1.22 (1.06 - 1.39)	0.0042	44.7 ± 1.2	1.16 (1.03 - 1.31)	0.0151
Vietnamese	39.7 ± 1.7	1.60 (1.39 - 1.85)	<0.0001	34.1 ± 1.6	1.56 (1.37 - 1.78)	<0.0001
White	36.8 ± 0.2	1.73 (1.54 - 1.95)	<0.0001	31.3 ± 0.2	1.71 (1.53 - 1.91)	<0.0001
Black	40.0 ± 0.5	1.63 (1.45 - 1.84)	<0.0001	32.6 ± 0.5	1.70 (1.52 - 1.90)	<0.0001
≥ 70 years						
South Asian	39.6 ± 3.3	1		28.1 ± 2.9	1	
Chinese	34.8 ± 1.1	1.13 (0.96 - 1.33)	0.1433	24.3 ± 1.0	1.10 (0.95 - 1.26)	0.1937
Filipino	37.4 ± 1.8	1.11 (0.93 - 1.32)	0.2420	23.8 ± 1.4	1.16 (1.00 - 1.34)	0.0532

**Japanese**	31.9 ± 1.1	1.26 (1.07 - 1.48)	0.0059	22.2 ± 0.9	1.22 (1.06 - 1.40)	0.0056
**Korean**	46.0 ± 1.3	0.84 (0.71 - 1.00)	0.0465	35.0 ± 1.2	0.84 (0.73 - 0.97)	0.0156
**Vietnamese**	32.9 ± 2.0	1.20 (1.00 - 1.43)	0.0500	24.5 ± 1.7	1.10 (0.94 - 1.28)	0.2196
**White**	28.1 ± 0.2	1.42 (1.22 - 1.66)	<0.0001	18.0 ± 0.2	1.39 (1.22 - 1.58)	<0.0001
**Black**	29.3 ± 0.6	1.41 (1.20 - 1.64)	<0.0001	17.4 ± 0.5	1.44 (1.26 - 1.64)	<0.0001
**Male**						
**South Asian**	50.4 ± 2.3	1		42.5 ± 2.2	1	
**Chinese**	38.8 ± 1.1	1.41 (1.23 - 1.60)	<0.0001	30.1 ± 1.0	1.42 (1.27 - 1.60)	<0.0001
**Filipino**	33.6 ± 1.6	1.65 (1.43 - 1.90)	<0.0001	24.4 ± 1.4	1.71 (1.51 - 1.94)	<0.0001
**Japanese**	34.4 ± 1.3	1.67 (1.46 - 1.91)	<0.0001	25.1 ± 1.1	1.78 (1.59 - 2.01)	<0.0001
**Korean**	48.3 ± 1.2	1.08 (0.95 - 1.24)	0.2381	40.2 ± 1.1	1.12 (0.99 - 1.25)	0.0716
**Vietnamese**	36.0 ± 1.7	1.52 (1.32 - 1.76)	<0.0001	42.5 ± 2.2	1.45 (1.27 - 1.65)	<0.0001
**White**	29.6 ± 0.2	1.85 (1.64 - 2.09)	<0.0001	22.1 ± 0.2	1.85 (1.66 - 2.06)	<0.0001
**Black**	31.1 ± 0.5	1.83 (1.62 - 2.07)	<0.0001	22.1 ± 0.4	1.89 (1.70 - 2.11)	<0.0001
**Female**						
**South Asian**	46.8 ± 1.4	1		45.1 ± 2.8	1	

**Chinese**	40.3 ± 1.3	1.35 (1.15 - 1.59)	0.0003	32.8 ± 1.2	1.35 (1.16 - 1.55)	<0.0001
**Filipino**	40.5 ± 1.7	1.33 (1.12 - 1.58)	0.0011	32.1 ± 1.6	1.37 (1.17 - 1.59)	<0.0001
**Japanese**	36.3 ± 1.4	1.50 (1.28 - 1.77)	<0.0001	27.8 ± 1.2	1.58 (1.37 - 1.83)	<0.0001
**Korean**	46.8 ± 1.4	1.11 (0.94 - 1.31)	0.2060	40.1 ± 1.3	1.09 (0.94 - 1.26)	0.2593
**Vietnamese**	37.9 ± 2.1	1.43 (1.19 - 1.71)	0.0001	30.4 ± 1.9	1.45 (1.23 - 1.70)	<0.0001
**White**	37.6 ± 0.3	1.48 (1.27 - 1.73)	<0.0001	29.0 ± 0.3	1.53 (1.34 - 1.75)	<0.0001
**Black**	41.4 ± 0.6	1.33 (1.14 - 1.55)	0.0002	31.4 ± 0.6	1.43 (1.25 - 1.64)	<0.0001
**Localized-stage**						
**South Asian**	86.9 ± 2.1	1	1	79.8 ± 2.5	1	
**Chinese**	75.9 ± 1.5	1.98 (1.42 - 2.75)	<0.0001	62.3 ± 1.6	2.07 (1.63 - 2.63)	<0.0001
**Filipino**	77.1 ± 2.0	1.76 (1.23 - 2.50)	0.0018	61.8 ± 2.2	2.03 (1.57 - 2.62)	<0.0001
**Japanese**	69.7 ± 1.6	2.61 (1.88 - 3.62)	<0.0001	52.9 ± 1.7	2.94 (2.32 - 3.73)	<0.0001
**Korean**	83.8 ± 1.1	1.26 (0.90 - 1.76)	0.1710	73.3 ± 1.3	1.45 (1.14 - 1.85)	0.0025
**Vietnamese**	73.2 ± 2.5	2.12 (1.48 - 3.04)	<0.0001	64.0 ± 2.6	1.91 (1.46 - 2.49)	<0.0001
**White**	69.2 ± 0.3	2.56 (1.89 - 3.49)	<0.0001	53.6 ± 0.3	2.67 (2.13 - 3.35)	<0.0001
**Black**	70.1 ± 0.7	2.50 (1.83 - 3.41)	<0.0001	53.1 ± 0.7	2.71 (2.16 - 3.41)	<0.0001

**Regional-stage**						
**South Asian**	45.9 ± 3.7	1		38.5 ± 3.5	1	
**Chinese**	42.5 ± 1.5	1.10 (0.90 - 1.33)	0.3526	34.7 ± 1.4	1.07 (0.90 - 1.27)	0.4635
**Filipino**	39.3 ± 2.3	1.17 (0.94 - 1.44)	0.1544	30.2 ± 2.1	1.20 (1.00 - 1.44)	0.0562
**Japanese**	36.3 ± 1.7	1.31 (1.08 - 1.59)	0.0074	29.0 ± 1.5	1.34 (1.12 - 1.59)	0.0011
**Korean**	45.5 ± 1.6	0.96 (0.79 - 1.17)	0.6775	38.8 ± 1.5	0.94 (0.79 - 1.11)	0.4466
**Vietnamese**	42.4 ± 2.3	1.14 (0.92 - 1.41)	0.2244	36.0 ± 2.2	1.08 (0.89 - 1.30)	0.4533
**White**	31.1 ± 0.3	1.48 (1.24 - 1.78)	<0.0001	24.5 ± 0.3	1.43 (1.22 - 1.68)	<0.0001
**Black**	33.8 ± 0.8	1.41 (1.18 - 1.70)	0.0002	26.2 ± 0.7	1.40 (1.19 - 1.64)	<0.0001
**Distant-stage**						
**South Asian**	11.2 ± 2.3	1		8.5 ± 1.9	1	
**Chinese**	9.4 ± 1.0	1.22 (1.06 - 1.40)	0.0052	7.5 ± 0.8	1.18 (1.04 - 1.35)	0.013
**Filipino**	7.5 ± 1.2	1.34 (1.16 - 1.56)	<0.0001	5.3 ± 0.9	1.36 (1.18 - 1.56)	<0.0001
**Japanese**	5.7 ± 0.9	1.46 (1.26 - 1.68)	<0.0001	3.9 ± 0.7	1.43 (1.25 - 1.63)	<0.0001
**Korean**	6.3 ± 0.9	1.32 (1.15 - 1.53)	0.0001	5.0 ± 0.8	1.27 (1.11 - 1.46)	0.0005
**Vietnamese**	8.2 ± 1.4	1.22 (1.05 - 1.42)	0.0108	6.0 ± 1.1	1.23 (1.06 - 1.42)	0.0054

**White**	6.4 ± 0.2	1.42 (1.25 - 1.62)	<0.0001	4.8 ± 0.1	1.40 (1.24 - 1.57)	<0.0001
**Black**	8.0 ± 0.4	1.39 (1.22 - 1.58)	<0.0001	5.5 ± 0.3	1.39 (1.23 - 1.57)	<0.0001
**No surgery**						
**South Asian**	28.9 ± 2.5	1		21.2 ± 2.1	1	
**Chinese**	13.3 ± 1.0	1.52 (1.34 - 1.72)	<0.0001	8.6 ± 0.7	1.48 (1.32 - 1.66)	<0.0001
**Filipino**	12.0 ± 1.3	1.65 (1.45 - 1.89)	<0.0001	6.9 ± 0.9	1.64 (1.45 - 1.85)	<0.0001
**Japanese**	7.8 ± 0.9	1.96 (1.73 - 2.23)	<0.0001	4.4 ± 0.6	1.91 (1.70 - 2.14)	<0.0001
**Korean**	12.9 ± 1.1	1.55 (1.36 - 1.77)	<0.0001	8.7 ± 0.9	1.49 (1.32 - 1.68)	<0.0001
**Vietnamese**	12.8 ± 1.5	1.55 (1.35 - 1.79)	<0.0001	7.8 ± 1.1	1.53 (1.35 - 1.74)	<0.0001
**White**	12.7 ± 0.2	1.67 (1.49 - 1.87)	<0.0001	8.1 ± 0.1	1.62 (1.46 - 1.80)	<0.0001
**Black**	14.1 ± 0.4	1.64 (1.46 - 1.84)	<0.0001	8.4 ± 0.3	1.66 (1.49 - 1.84)	<0.0001
**Surgery**						
**South Asian**	69.4 ± 2.3	1		63.8 ± 2.4	1	
**Chinese**	57.7 ± 1.1	1.54 (1.30 - 1.83)	<0.0001	48.4 ± 1.1	1.61 (1.39 - 1.87)	<0.0001
**Filipino**	59.6 ± 1.7	1.40 (1.16 - 1.69)	0.0005	49.4 ± 1.7	1.57 (1.34 - 1.85)	<0.0001
**Japanese**	55.4 ± 1.3	1.64 (1.38 - 1.96)	<0.0001	43.9 ± 1.2	1.96 (1.69 - 2.28)	<0.0001

**Korean**	65.5 ± 1.1	1.13 (0.95 - 1.35)	0.1776	57.9 ± 1.1	1.22 (1.05 - 1.41)	0.0112
**Vietnamese**	53.8 ± 1.8	1.72 (1.42 - 2.07)	<0.0001	47.5 ± 1.7	1.65 (1.40 - 1.94)	<0.0001
**White**	55.1 ± 0.3	1.63 (1.39 - 1.92)	<0.0001	45.1 ± 0.3	1.79 (1.56 - 2.06)	<0.0001
**Black**	57.5 ± 0.6	1.54 (1.30 - 1.81)	<0.0001	46.2 ± 0.6	1.75 (1.52 - 2.01)	<0.0001
**No chemotherapy**						
**South Asian**	66.0 ± 2.3	1		54.3 ± 2.4	1	
**Chinese**	43.0 ± 1.1	1.91 (1.62 - 2.25)	<0.0001	31.6 ± 1.0	1.79 (1.56 - 2.04)	<0.0001
**Filipino**	40.7 ± 1.7	2.08 (1.75 - 2.48)	<0.0001	28.7 ± 1.4	2.00 (1.73 - 2.31)	<0.0001
**Japanese**	39.0 ± 1.2	2.19 (1.85 - 2.58)	<0.0001	26.7 ± 1.0	2.14 (1.87 - 2.44)	<0.0001
**Korean**	53.6 ± 1.1	1.42 (1.20 - 1.68)	<0.0001	43.8 ± 1.1	1.32 (1.15 - 1.51)	<0.0001
**Vietnamese**	39.3 ± 1.8	2.09 (1.75 - 2.49)	<0.0001	31.2 ± 1.6	1.82 (1.57 - 2.11)	<0.0001
**White**	39.2 ± 0.2	2.22 (1.90 - 2.60)	<0.0001	27.9 ± 0.2	2.08 (1.83 - 2.36)	<0.0001
**Black**	39.6 ± 0.5	2.19 (1.87 - 2.56)	<0.0001	27.0 ± 0.5	2.12 (1.86 - 2.41)	<0.0001
**Chemotherapy**						
**South Asian**	37.0 ± 2.5	1		32.9 ± 2.4	1	
**Chinese**	35.5 ± 1.3	1.04 (0.91 - 1.19)	0.5609	30.7 ± 1.2	1.04 (0.92 - 1.18)	0.5048

**Filipino**	33.6 ± 1.7	1.12 (0.97 - 1.29)	0.1327	27.4 ± 1.5	1.16 (1.02 - 1.33)	0.0287
**Japanese**	29.3 ± 1.5	1.21 (1.05 - 1.38)	0.0082	25.2 ± 1.4	1.25 (1.10 - 1.43)	0.0006
**Korean**	38.6 ± 1.4	0.93 (0.81 - 1.06)	0.2726	34.0 ± 1.4	0.93 (0.82 - 1.06)	0.2888
**Vietnamese**	34.1 ± 1.9	1.10 (0.95 - 1.28)	0.2058	28.2 ± 1.7	1.13 (0.99 - 1.30)	0.0789
**White**	25.0 ± 0.2	1.39 (1.24 - 1.57)	<0.0001	20.3 ± 0.2	1.43 (1.28 - 1.60)	<0.0001
**Black**	30.8 ± 0.6	1.20 (1.06 - 1.35)	0.0035	24.8 ± 0.6	1.26 (1.13 - 1.42)	<0.0001
**No radiotherapy**						
**South Asian**	54.8 ± 2.0	1		46.7 ± 2.0	1	
**Chinese**	40.0 ± 1.0	1.51 (1.34 - 1.70)	<0.0001	31.2 ± 0.9	1.50 (1.35 - 1.66)	<0.0001
**Filipino**	38.2 ± 1.3	1.61 (1.42 - 1.83)	<0.0001	28.7 ± 1.2	1.63 (1.46 - 1.82)	<0.0001
**Japanese**	35.7 ± 1.1	1.75 (1.55 - 1.98)	<0.0001	25.7 ± 0.9	1.84 (1.65 - 2.04)	<0.0001
**Korean**	48.1 ± 1.0	1.21 (1.07 - 1.36)	0.0022	40.0 ± 0.9	1.20 (1.08 - 1.33)	0.0008
**Vietnamese**	36.9 ± 1.5	1.63 (1.43 - 1.86)	<0.0001	29.8 ± 1.3	1.57 (1.40 - 1.76)	<0.0001
**White**	34.3 ± 0.2	1.84 (1.65 - 2.05)	<0.0001	25.7 ± 0.2	1.82 (1.66 - 2.01)	<0.0001
**Black**	37.1 ± 0.5	1.71 (1.53 - 1.91)	<0.0001	26.9 ± 0.4	1.76 (1.60 - 1.95)	<0.0001
**Radiotherapy**						

**South Asian**	38.6 ± 3.7	1		33.3 ± 3.5	1	
**Chinese**	38.2 ± 1.8	1.01 (0.83 - 1.24)	0.8992	32.0 ± 1.7	1.04 (0.87 - 1.26)	0.6632
**Filipino**	33.1 ± 2.5	1.18 (0.95 - 1.46)	0.1424	26.0 ± 2.3	1.26 (1.03 - 1.54)	0.0229
**Japanese**	34.1 ± 2.0	1.15 (0.94 - 1.41)	0.1737	28.7 ± 1.8	1.25 (1.03 - 1.51)	0.0212
**Korean**	46.6 ± 2.1	0.77 (0.63 - 0.95)	0.0147	41.6 ± 2.0	0.80 (0.66 - 0.97)	0.0213
**Vietnamese**	37.0 ± 2.7	1.05 (0.84 - 1.32)	0.6428	31.0 ± 2.5	1.08 (0.88 - 1.32)	0.4865
**White**	27.7 ± 0.3	1.33 (1.11 - 1.59)	0.002	21.8 ± 0.3	1.42 (1.20 - 1.68)	<0.0001
**Black**	29.8 ± 0.9	1.27 (1.05 - 1.53)	0.013	23.0 ± 0.8	1.37 (1.15 - 1.63)	0.0004
**Cardia cancer**						
**South Asian**	30.8 ± 3.6	1		25.5 ± 3.3	1	
**Chinese**	33.7 ± 2.4	1.03 (0.84 - 1.26)	0.7811	25.0 ± 2.1	1.12 (0.93 - 1.35)	0.2378
**Filipino**	27.6 ± 2.4	1.16 (0.95 - 1.43)	0.1468	21.4 ± 2.1	1.22 (1.01 - 1.47)	0.0402
**Japanese**	27.9 ± 2.4	1.16 (0.95 - 1.43)	0.1458	20.4 ± 2.0	1.27 (1.06 - 1.53)	0.0112
**Korean**	30.2 ± 3.7	0.97 (0.76 - 1.24)	0.8053	24.6 ± 3.2	1.03 (0.82 - 1.28)	0.8161
**Vietnamese**	19.2 ± 3.6	1.34 (1.05 - 1.72)	0.0203	15.7 ± 3.2	1.32 (1.05 - 1.67)	0.0191
**White**	25.5 ± 0.3	1.25 (1.06 - 1.48)	0.0092	19.3 ± 0.2	1.31 (1.12 - 1.54)	0.0007

**Black**	24.5 ± 1.1	1.34 (1.13 - 1.60)	0.0011	17.3 ± 0.9	1.45 (1.23 - 1.71)	<0.0001
**Non-cardia cancer**						
**South Asian**	57.1 ± 2.0	1		48.9 ± 2.0	1	
**Chinese**	40.3 ± 0.9	1.57 (1.39 - 1.77)	<0.0001	32.1 ± 0.8	1.52 (1.37 - 1.69)	<0.0001
**Filipino**	39.6 ± 1.4	1.64 (1.44 - 1.86)	<0.0001	29.9 ± 1.2	1.66 (1.48 - 1.86)	<0.0001
**Japanese**	36.5 ± 1.0	1.81 (1.60 - 2.04)	<0.0001	27.2 ± 0.9	1.87 (1.68 - 2.07)	<0.0001
**Korean**	48.6 ± 0.9	1.25 (1.11 - 1.41)	0.0004	41.1 ± 0.9	1.22 (1.09 - 1.35)	0.0003
**Vietnamese**	38.6 ± 1.4	1.65 (1.45 - 1.88)	<0.0001	31.3 ± 1.3	1.57 (1.40 - 1.76)	<0.0001
**White**	36.4 ± 0.2	1.84 (1.64 - 2.06)	<0.0001	27.5 ± 0.2	1.84 (1.66 - 2.03)	<0.0001
**Black**	37.2 ± 0.4	1.77 (1.58 - 1.98)	<0.0001	27.4 ± 0.4	1.81 (1.64 - 2.00)	<0.0001
**Diffuse histology**						
**South Asian**	29.6 ± 3.7	1		24.0 ± 3.3	1	
**Chinese**	31.3 ± 1.6	1.02 (0.84 - 1.23)	0.8603	25.6 ± 1.5	1.01 (0.85 - 1.20)	0.922
**Filipino**	25.0 ± 2.1	1.19 (0.97 - 1.45)	0.0998	19.4 ± 1.8	1.21 (1.00 - 1.45)	0.051
**Japanese**	29.7 ± 1.9	1.12 (0.92 - 1.37)	0.2497	23.6 ± 1.6	1.17 (0.98 - 1.40)	0.0901
**Korean**	42.4 ± 1.7	0.75 (0.61 - 0.91)	0.0031	36.8 ± 1.6	0.74 (0.62 - 0.88)	0.0009

**Vietnamese**	28.6 ± 2.4	1.10 (0.89 - 1.35)	0.3890	23.5 ± 2.1	1.10 (0.91 - 1.33)	0.3436
**White**	19.3 ± 0.3	1.45 (1.22 - 1.73)	<0.0001	14.9 ± 0.3	1.44 (1.22 - 1.69)	<0.0001
**Black**	20.5 ± 0.8	1.41 (1.18 - 1.69)	0.0002	15.0 ± 0.7	1.44 (1.22 - 1.71)	<0.0001
**Non-diffuse histology**						
**South Asian**	56.6 ± 2.0	1		41.4 ± 1.0	1	
**Chinese**	42.2 ± 1.0	1.51 (1.34 - 1.70)	<0.0001	33.1 ± 0.9	1.52 (1.37 - 1.69)	<0.0001
**Filipino**	41.3 ± 1.4	1.57 (1.38 - 1.79)	<0.0001	31.2 ± 1.3	1.63 (1.45 - 1.82)	<0.0001
**Japanese**	36.9 ± 1.1	1.77 (1.57 - 2.00)	<0.0001	27.1 ± 0.9	1.88 (1.69 - 2.09)	<0.0001
**Korean**	49.6 ± 1.0	1.22 (1.08 - 1.38)	0.0017	41.4 ± 1.0	1.24 (1.11 - 1.38)	<0.0001
**Vietnamese**	39.9 ± 1.5	1.60 (1.40 - 1.83)	<0.0001	32.3 ± 1.4	1.55 (1.37 - 1.74)	<0.0001
**White**	36.0 ± 0.2	1.81 (1.62 - 2.03)	<0.0001	27.2 ± 0.2	1.83 (1.66 - 2.02)	<0.0001
**Black**	38.8 ± 0.5	1.71 (1.53 - 1.92)	<0.0001	28.5 ± 0.4	1.79 (1.62 - 1.98)	<0.0001

*

Table S5. **Characteristics of Asian American, white, and black patients diagnosed with gastric cancer during 2000-2010.**

Variable	Overall	Chinese	Korean	Japanese	Filipino	Vietnamese	South Asian	White	Black	P value
	62109 (100)	1963 (3)	1877 (3)	1938 (3)	979 (2)	787 (1)	347 (1)	45863 (74)	8355 (13)	
**Age (year)**	69684 (100)	2377 (3)	1910 (3)	1269 (2)	1194 (2)	988 (1)	692 (1)	51544 (74)	9710 (14)	
**Mean ± SD**	68.2 ± 14.1	69.6 ± 14.4	65.5 ± 13.5	74.4 ± 11.5	67.7 ± 13.9	65.7 ± 14.8	60.3 ± 15.1	68.4 ± 14.1	66.3 ± 14.3	<0.0001
**Group**										<0.0001
**<50**	6949 (11)	217 (11)	253 (13)	72 (4)	99 (10)	137 (17)	89 (26)	4955 (11)	1127 (13)	
**50- 59**	9550 (15)	246 (13)	333 (18)	164 (8)	177 (18)	134 (17)	79 (23)	6908 (15)	1509 (18)	
**60-69**	13593 (22)	377 (19)	502 (27)	285 (15)	206 (21)	166 (21)	77 (22)	10019 (22)	1961 (23)	
**70-79**	16918 (27)	557 (28)	509 (27)	702 (36)	287 (29)	184 (23)	65 (19)	12512 (27)	2102 (25)	
**≥80**	15099 (24)	566 (29)	280 (15)	715 (37)	210 (21)	166 (21)	37 (11)	11469 (25)	1656 (20)	
**Gender**										<0.0001
**Male**	24400 (39)	847 (43)	807 (43)	868 (45)	474 (48)	331 (42)	130 (37)	17362 (38)	3581 (43)	
**Female**	37709 (61)	1116 (57)	1070 (57)	1070 (55)	505 (52)	456 (58)	217 (63)	28501 (62)	4774 (57)	
**Marital status**										<0.0001
**Married**	34790 (56)	1338 (68)	1274 (68)	1188 (61)	620 (63)	532 (68)	247 (71)	26385 (58)	3206 (38)	
**Unmarried**	24498 (39)	566 (29)	523 (28)	703 (36)	328 (34)	220 (28)	77 (22)	17376 (38)	4705 (56)	
**Unknown**	2821 (5)	59 (3)	80 (4)	47 (2)	31 (3)	35 (4)	23 (7)	2102 (5)	444 (5)	

**Region**										<0.0001
**West**	35203 (57)	1750 (89)	1591 (85)	1887 (97)	912 (93)	708 (90)	195 (56)	25713 (56)	2447 (29)	
**South**	12454 (20)	54 (3)	91 (5)	24 (1)	4 (0)	46 (6)	30 (9)	8022 (17)	4183 (50)	
**Midwest**	2022 (3)	2 (0)	3 (0)	5 (0)	0 (0)	10 (1)	2 (1)	1949 (4)	51 (1)	
**Northeast**	12430 (20)	157 (8)	192 (10)	22 (1)	63 (6)	23 (3)	120 (35)	10179 (22)	1674 (20)	
**Income ($)**										<0.0001
**<75,000**	4844 (30)	1180 (27)	1509 (40)	914 (29)	563 (26)	411 (23)	267 (26)	44469 (46)	10782 (60)	
**75,000-94,999**	5350 (33)	1102 (25)	1111 (29)	1505 (47)	857 (39)	488 (27)	287 (28)	30346 (31)	4860 (27)	
**≥ 95,000**	6127 (38)	2058 (47)	1167 (31)	788 (25)	753 (35)	876 (49)	485 (47)	22592 (23)	2423 (13)	
**Primary site**										<0.0001
**Overlapping or not specified**	15900 (26)	458 (23)	450 (24)	459 (24)	261 (27)	191 (24)	101 (29)	11472 (25)	2508 (30)	
**Antrum**	11476 (18)	643 (33)	646 (34)	534 (28)	216 (22)	263 (33)	63 (18)	7105 (15)	2006 (24)	
**Body**	5817 (9)	148 (8)	227 (12)	267 (14)	93 (10)	70 (9)	35 (10)	4133 (9)	844 (10)	
**Cardia**	16684 (27)	224 (11)	82 (4)	250 (13)	174 (18)	73 (9)	75 (22)	14819 (32)	987 (12)	
**Fundus**	2970 (5)	85 (4)	53 (3)	60 (3)	48 (5)	16 (2)	20 (6)	2242 (5)	446 (5)	
**Greater curvature**	2627 (4)	112 (6)	86 (5)	86 (4)	58 (6)	47 (6)	12 (3)	1826 (4)	400 (5)	
**Lesser curvature**	4916 (8)	235 (12)	262 (14)	232 (12)	100 (10)	93 (12)	32 (9)	3144 (7)	818 (10)	
**Pylorus**	1719 (3)	58 (3)	71 (4)	50 (3)	29 (3)	34 (4)	9 (3)	1122 (2)	346 (4)	

**Tumor size (cm)**										<0.0001
**≤ 3**	7542 (12)	298 (15)	356 (19)	251 (13)	123 (13)	115 (15)	68 (20)	5475 (12)	856 (10)	
**>3**	21971 (35)	795 (41)	725 (39)	857 (44)	411 (42)	332 (42)	107 (31)	15597 (34)	3147 (38)	
**Unknown**	32596 (52)	870 (44)	796 (42)	830 (43)	445 (45)	340 (43)	172 (50)	24791 (54)	4352 (52)	
**Stage**										<0.0001
**Localized**	15857 (26)	481 (25)	585 (31)	563 (29)	235 (24)	160 (20)	96 (28)	11502 (25)	2235 (27)	
**Regional**	17420 (28)	679 (35)	649 (35)	621 (32)	297 (30)	278 (35)	94 (27)	12532 (27)	2270 (27)	
**Distant**	21442 (35)	572 (29)	467 (25)	584 (30)	351 (36)	274 (35)	115 (33)	16267 (35)	2812 (34)	
**Unknown**	7390 (12)	231 (12)	176 (9)	170 (9)	96 (10)	75 (10)	42 (12)	5562 (12)	1038 (12)	
**Histology**										<0.0001
**Diffuse**	13239 (21)	495 (25)	505 (27)	458 (24)	255 (26)	220 (28)	90 (26)	9682 (21)	1534 (18)	
**Intestinal**	37657 (61)	1186 (60)	1175 (63)	1226 (63)	530 (54)	473 (60)	158 (46)	27852 (61)	5057 (61)	
**Other**	11213 (18)	282 (14)	197 (11)	254 (13)	194 (20)	94 (12)	99 (29)	8329 (18)	1764 (21)	
**Grade**										<0.0001
**I**	2785 (4)	56 (3)	67 (4)	95 (5)	28 (3)	21 (3)	24 (7)	2082 (5)	412 (5)	
**II**	12806 (21)	381 (19)	414 (22)	438 (23)	184 (19)	156 (20)	60 (17)	9334 (20)	1839 (22)	
**III/IV**	32324 (52)	1142 (58)	1131 (60)	1150 (59)	548 (56)	489 (62)	163 (47)	23711 (52)	3990 (48)	
**Unknown**	14194 (23)	384 (20)	265 (14)	255 (13)	219 (22)	121 (15)	100 (29)	10736 (23)	2114 (25)	

**Surgery**										<0.0001
**No**	31458 (51)	770 (39)	586 (31)	776 (40)	464 (47)	305 (39)	164 (47)	24120 (53)	4273 (51)	
**Yes**	30651 (49)	1193 (61)	1291 (69)	1162 (60)	515 (53)	482 (61)	183 (53)	21743 (47)	4082 (49)	
**Chemotherapy**										<0.0001
**No**	39073 (63)	1247 (64)	1216 (65)	1300 (67)	595 (61)	485 (62)	195 (56)	28420 (62)	5615 (67)	
**Yes**	23036 (37)	716 (36)	661 (35)	638 (33)	384 (39)	302 (38)	152 (44)	17443 (38)	2740 (33)	
**Radiotherapy**										<0.0001
**No**	48819 (79)	1535 (78)	1485 (79)	1523 (79)	766 (78)	602 (76)	266 (77)	35706 (78)	6936 (83)	
**Yes**	13290 (21)	428 (22)	392 (21)	415 (21)	213 (22)	185 (24)	81 (23)	10157 (22)	1419 (17)	
**Cancer specific death**										<0.0001
**No**	20396 (33)	790 (40)	842 (45)	713 (37)	382 (39)	277 (35)	164 (47)	14427 (31)	2801 (34)	
**Yes**	41713 (67)	1173 (60)	1035 (55)	1225 (63)	597 (61)	510 (65)	183 (53)	31436 (69)	5554 (66)	
**Overall death**										<0.0001
**No**	7440 (12)	391 (20)	459 (24)	198 (10)	152 (16)	132 (17)	110 (32)	5091 (11)	907 (11)	7440 (12)
**Yes**	54669 (88)	1572 (80)	1418 (76)	1740 (90)	827 (84)	655 (83)	237 (68)	40772 (89)	7448 (89)	54669 (88)

Table S6. **Characteristics of Asian American, white, and black patients with gastric cancer diagnosed during 2011-2021**.

Variable	Overall	Chinese	Korean	Japanese	Filipino	Vietnamese	South Asian	White	Black	P value
	69684 (100)	2377 (3)	1910 (3)	1269 (2)	1194 (2)	988 (1)	692 (1)	51544 (74)	9710 (14)	
**Age (year)**										
**Mean ± SD**	67.2 ± 13.8	69.6 ± 13.9	68.9 ± 12.3	75.8 ± 12.2	67.7 ± 13.2	65.8 ± 14.2	62.2 ± 14.6	67.2 ± 13.8	65.6 ± 13.4	<0.0001
**Group**										<0.0001
**<50**	7489 (11)	222 (9)	141 (7)	42 (3)	106 (9)	133 (13)	140 (20)	5582 (11)	1123 (12)	
**50- 59**	11555 (17)	300 (13)	275 (14)	92 (7)	169 (14)	176 (18)	132 (19)	8477 (16)	1934 (20)	
**60-69**	18289 (26)	564 (24)	499 (26)	220 (17)	355 (30)	247 (25)	184 (27)	13452 (26)	2768 (29)	
**70-79**	18107 (26)	633 (27)	594 (31)	318 (25)	342 (29)	258 (26)	155 (22)	13503 (26)	2304 (24)	
**≥80**	14244 (20)	658 (28)	401 (21)	597 (47)	222 (19)	174 (18)	81 (12)	10530 (20)	1581 (16)	
**Gender**										<0.0001
**Male**	27726 (40)	1054 (44)	749 (39)	614 (48)	575 (48)	387 (39)	264 (38)	19778 (38)	4305 (44)	
**Female**	41958 (60)	1323 (56)	1161 (61)	655 (52)	619 (52)	601 (61)	428 (62)	31766 (62)	5405 (56)	
**Marital status**										<0.0001
**Married**	38026 (55)	1656 (70)	1272 (67)	662 (52)	741 (62)	647 (65)	495 (72)	28860 (56)	3693 (38)	
**Unmarried**	27324 (39)	612 (26)	520 (27)	528 (42)	409 (34)	291 (29)	154 (22)	19539 (38)	5271 (54)	
**Unknown**	4334 (6)	109 (5)	118 (6)	79 (6)	44 (4)	50 (5)	43 (6)	3145 (6)	746 (8)	

**Region**										<0.0001
**West**	39650 (57)	2116 (89)	1497 (78)	1214 (96)	1095 (92)	879 (89)	356 (51)	29736 (58)	2757 (28)	
**South**	15408 (22)	59 (2)	151 (8)	21 (2)	13 (1)	67 (7)	91 (13)	10036 (19)	4970 (51)	
**Midwest**	1986 (3)	5 (0)	2 (0)	5 (0)	3 (0)	12 (1)	4 (1)	1886 (4)	69 (1)	
**Northeast**	12640 (18)	197 (8)	260 (14)	29 (2)	83 (7)	30 (3)	241 (35)	9886 (19)	1914 (20)	
**Income ($)**										<0.0001
**<75,000**	21710 (31)	218 (9)	229 (12)	164 (13)	120 (10)	117 (12)	94 (14)	16291 (32)	4477 (46)	
**75,000-94,999**	23837 (34)	704 (30)	818 (43)	313 (25)	408 (34)	270 (27)	201 (29)	17986 (35)	3137 (32)	
**≥ 95,000**	24137 (35)	1455 (61)	863 (45)	792 (62)	666 (56)	601 (61)	397 (57)	17267 (34)	2096 (22)	
**Primary site**										<0.0001
**Overlapping or NOS**	17448 (25)	544 (23)	453 (24)	306 (24)	305 (26)	238 (24)	175 (25)	12558 (24)	2869 (30)	
**Antrum**	10738 (15)	632 (27)	598 (31)	316 (25)	185 (15)	295 (30)	98 (14)	6596 (13)	2018 (21)	
**Body**	8081 (12)	291 (12)	270 (14)	176 (14)	141 (12)	111 (11)	121 (17)	5721 (11)	1250 (13)	
**Cardia**	20433 (29)	304 (13)	137 (7)	204 (16)	276 (23)	97 (10)	158 (23)	17947 (35)	1310 (13)	
**Fundus**	3619 (5)	118 (5)	63 (3)	45 (4)	79 (7)	40 (4)	38 (5)	2622 (5)	614 (6)	
**Greater curvature**	3061 (4)	117 (5)	68 (4)	59 (5)	89 (7)	50 (5)	37 (5)	2074 (4)	567 (6)	
**Lesser curvature**	4729 (7)	279 (12)	264 (14)	139 (11)	93 (8)	107 (11)	52 (8)	3011 (6)	784 (8)	
**Pylorus**	1575 (2)	92 (4)	57 (3)	24 (2)	26 (2)	50 (5)	13 (2)	1015 (2)	298 (3)	

**Tumor size (cm)**										<0.0001
**≤ 3**	16527 (24)	528 (22)	627 (33)	326 (26)	252 (21)	230 (23)	179 (26)	12298 (24)	2087 (21)	
**>3**	23431 (34)	982 (41)	596 (31)	449 (35)	520 (44)	418 (42)	226 (33)	16547 (32)	3693 (38)	
**Unknown**	29726 (43)	867 (36)	687 (36)	494 (39)	422 (35)	340 (34)	287 (41)	22699 (44)	3930 (40)	
**Stage**										
**Localized**	21764 (31)	648 (27)	714 (37)	373 (29)	352 (29)	258 (26)	244 (35)	15866 (31)	3309 (34)	
**Regional**	16202 (23)	684 (29)	531 (28)	344 (27)	288 (24)	292 (30)	158 (23)	11802 (23)	2103 (22)	
**Distant**	24741 (36)	772 (32)	489 (26)	372 (29)	468 (39)	362 (37)	222 (32)	18749 (36)	3307 (34)	
**Unknown**	6977 (10)	273 (11)	176 (9)	180 (14)	86 (7)	76 (8)	68 (10)	5127 (10)	991 (10)	
**Histology**										<0.0001
**Diffuse**	12892 (19)	537 (23)	490 (26)	262 (21)	308 (26)	256 (26)	118 (17)	9507 (18)	1414 (15)	
**Intestinal**	39132 (56)	1406 (59)	1175 (62)	794 (63)	568 (48)	557 (56)	322 (47)	29196 (57)	5114 (53)	
**Other**	17660 (25)	434 (18)	245 (13)	213 (17)	318 (27)	175 (18)	252 (36)	12841 (25)	3182 (33)	
**Grade**										<0.0001
**I**	7794 (11)	176 (7)	125 (7)	98 (8)	101 (8)	53 (5)	117 (17)	5797 (11)	1327 (14)	
**II**	13714 (20)	467 (20)	393 (21)	273 (22)	205 (17)	188 (19)	112 (16)	10214 (20)	1862 (19)	
**III/IV**	32241 (46)	1291 (54)	1070 (56)	648 (51)	607 (51)	569 (58)	313 (45)	23781 (46)	3962 (41)	
**Unknown**	15935 (23)	443 (19)	322 (17)	250 (20)	281 (24)	178 (18)	150 (22)	11752 (23)	2559 (26)	

**Surgery**										<0.0001
**No**	39707 (57)	1167 (49)	823 (43)	679 (54)	654 (55)	506 (51)	345 (50)	30154 (59)	5379 (55)	
**Yes**	29977 (43)	1210 (51)	1087 (57)	590 (46)	540 (45)	482 (49)	347 (50)	21390 (42)	4331 (45)	
**Chemotherapy**										<0.0001
**No**	36245 (52)	1232 (52)	1086 (57)	768 (61)	559 (47)	473 (48)	341 (49)	26445 (51)	5341 (55)	
**Yes**	33439 (48)	1145 (48)	824 (43)	501 (39)	635 (53)	515 (52)	351 (51)	25099 (49)	4369 (45)	
**Radiotherapy**										<0.0001
**No**	55568 (80)	1969 (83)	1647 (86)	1039 (82)	974 (82)	809 (82)	564 (82)	40335 (78)	8231 (85)	
**Yes**	14116 (20)	408 (17)	263 (14)	230 (18)	220 (18)	179 (18)	128 (19)	11209 (22)	1479 (15)	
**Cancer specific death**										<0.0001
**No**	33585 (48)	1171 (49)	1089 (57)	545 (43)	586 (49)	488 (49)	440 (64)	24282 (47)	4984 (51)	
**Yes**	36099 (52)	1206 (51)	821 (43)	724 (57)	608 (51)	500 (51)	252 (36)	27262 (53)	4726 (49)	
**Overall death**										<0.0001
**No**	33585 (48)	1171 (49)	1089 (57)	545 (43)	586 (49)	488 (49)	440 (64)	24282 (47)	4984 (51)	
**Yes**	36099 (52)	1206 (51)	821 (43)	724 (57)	608 (51)	500 (51)	252 (36)	27262 (53)	4726 (49)	

Figure S1. Kaplan-Meier cancer-specific and overall survival curves among gastric cancer patients by racial/ethnic group after subgroup stratification. Cancer specific survival and overall survival curves are presented for patients aged <70 years (**A and B,** respectively), those with non-cardia gastric cancer (**C and D,** respectively), non-diffuse histology (**E and F,** respectively), and those who did not undergo surgery (**G and H,** respectively).

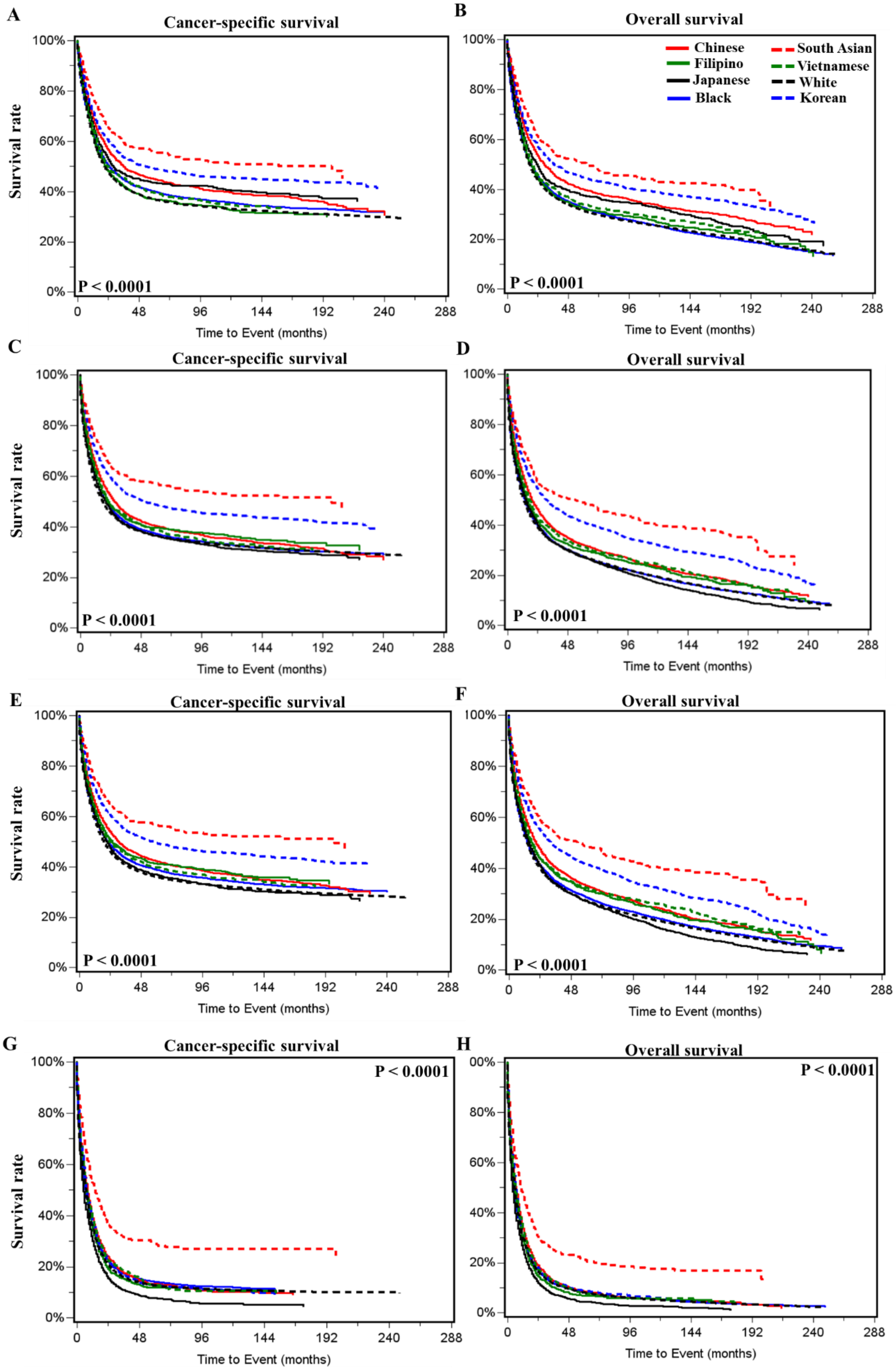
A
Cancer-specific survival
B
Overall survival
Chinese
Filipino
Japanese
Black
South Asian
Vietnamese
White
Korean
C
Cancer-specific survival
D
Overall survival
E
Cancer-specific survival
F
Overall survival
G
Cancer-specific survival
H
Overall survival
Survival rate
Time to Event (months)
P < 0.0001
100%
80%
60%
40%
20%
0%
0
48
96
144
192
240
288

Figure S2. Kaplan-Meier cancer-specific and overall survival curves among racial/ethnic groups of patients with gastric cancer after subgroup stratification. Cancer specific survival and overall survival curves are presented for patients who did not receive chemotherapy (**A and B,** respectively), those who did not receive radiotherapy (**C and D,** respectively), and patients with distant disease (**E and F,** respectively). Overall survival curves are additionally presented for patients with localized disease (**G**), and those who underwent surgery (**H**).

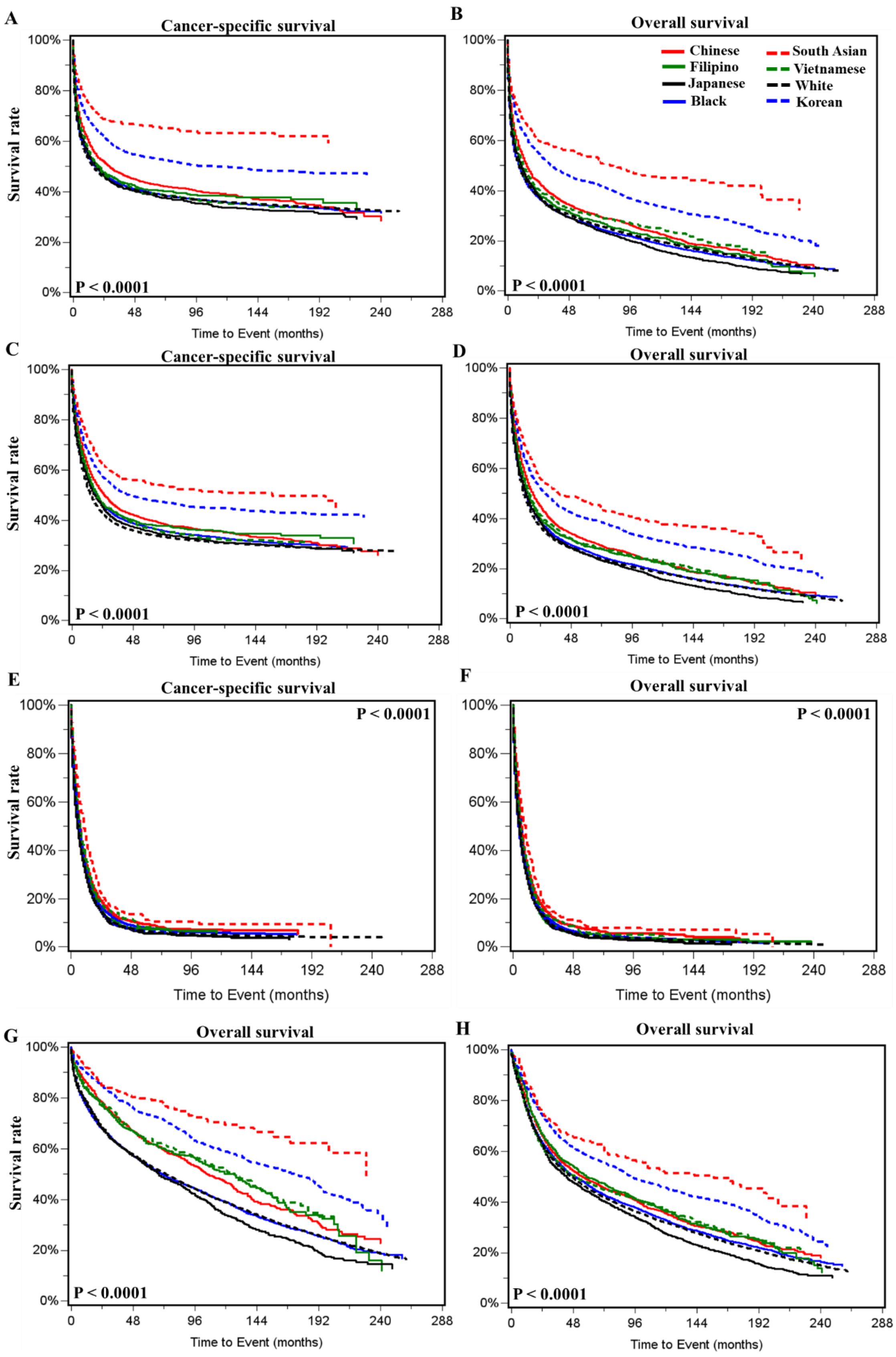
A
Cancer-specific survival
B
Overall survival
Chinese
Filipino
Japanese
Black
South Asian
Vietnamese
White
Korean
C
Cancer-specific survival
D
Overall survival
E
Cancer-specific survival
F
Overall survival
G
Overall survival
H
Overall survival
Survival rate
Time to Event (months)
P < 0.0001
100%
80%
60%
40%
20%
0%
0
48
96
144
192
240
288

Figure S3. Kaplan-Meier cancer-specific and overall survival curves among racial/ethnic groups of patients with gastric cancer after subgroup stratification. Cancer specific survival and overall survival curves are presented for males (**A and B,** respectively) and females (**C and D,** respectively). Cancer-specific survival curves are additionally presented for patients with localized tumors (**E**), and those who underwent surgery (**F**).

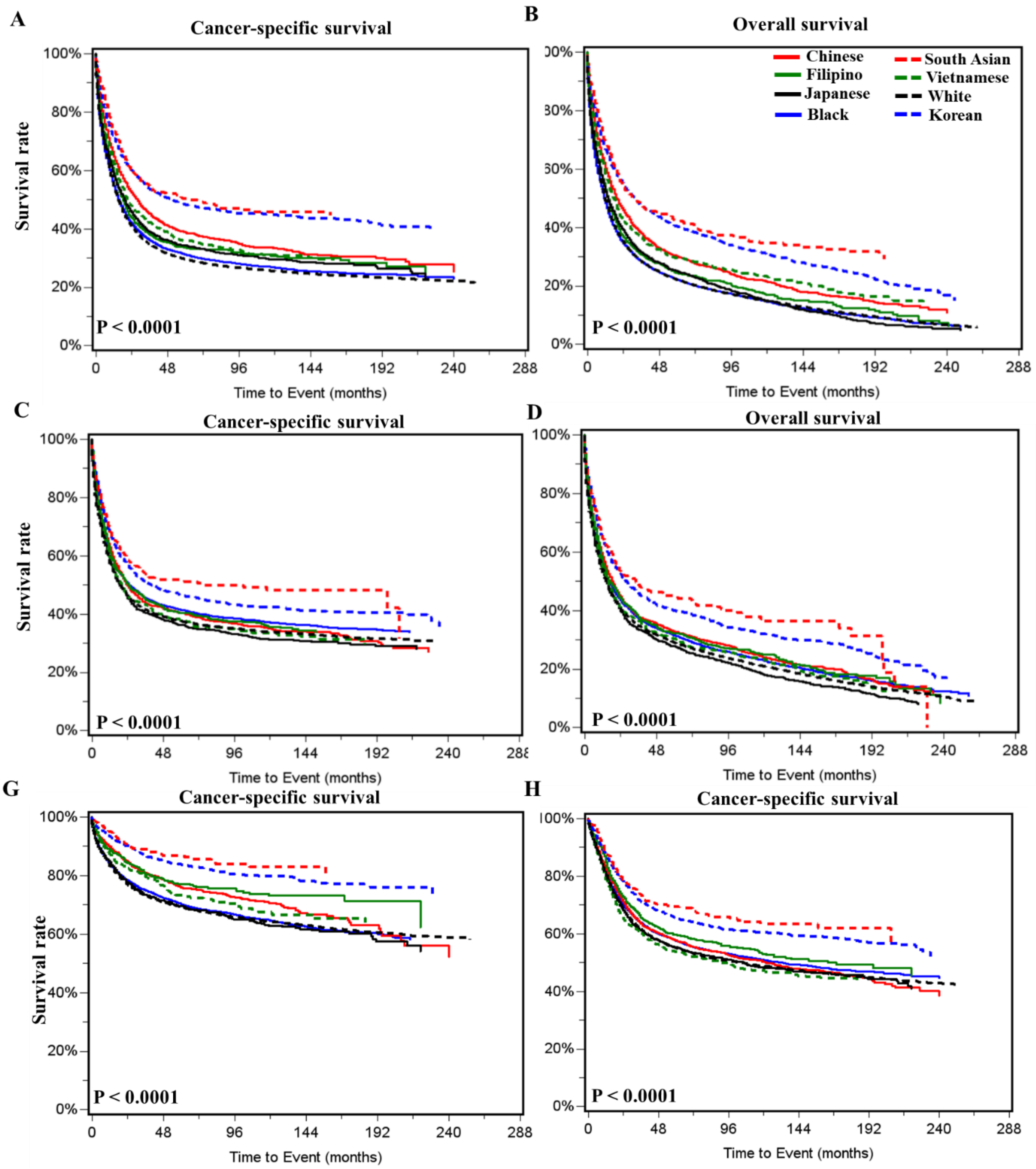

Figure S4. Kaplan-Meier cancer-specific and overall survival curves among racial/ethnic groups of patients with gastric cancer after subgroup stratification. Cancer specific survival and overall survival curves are presented for patients aged ≥ 70years (**A and B,** respectively), those with diffuse histology (**C and D,** respectively), and those who received radiotherapy (**E and F,** respectively).

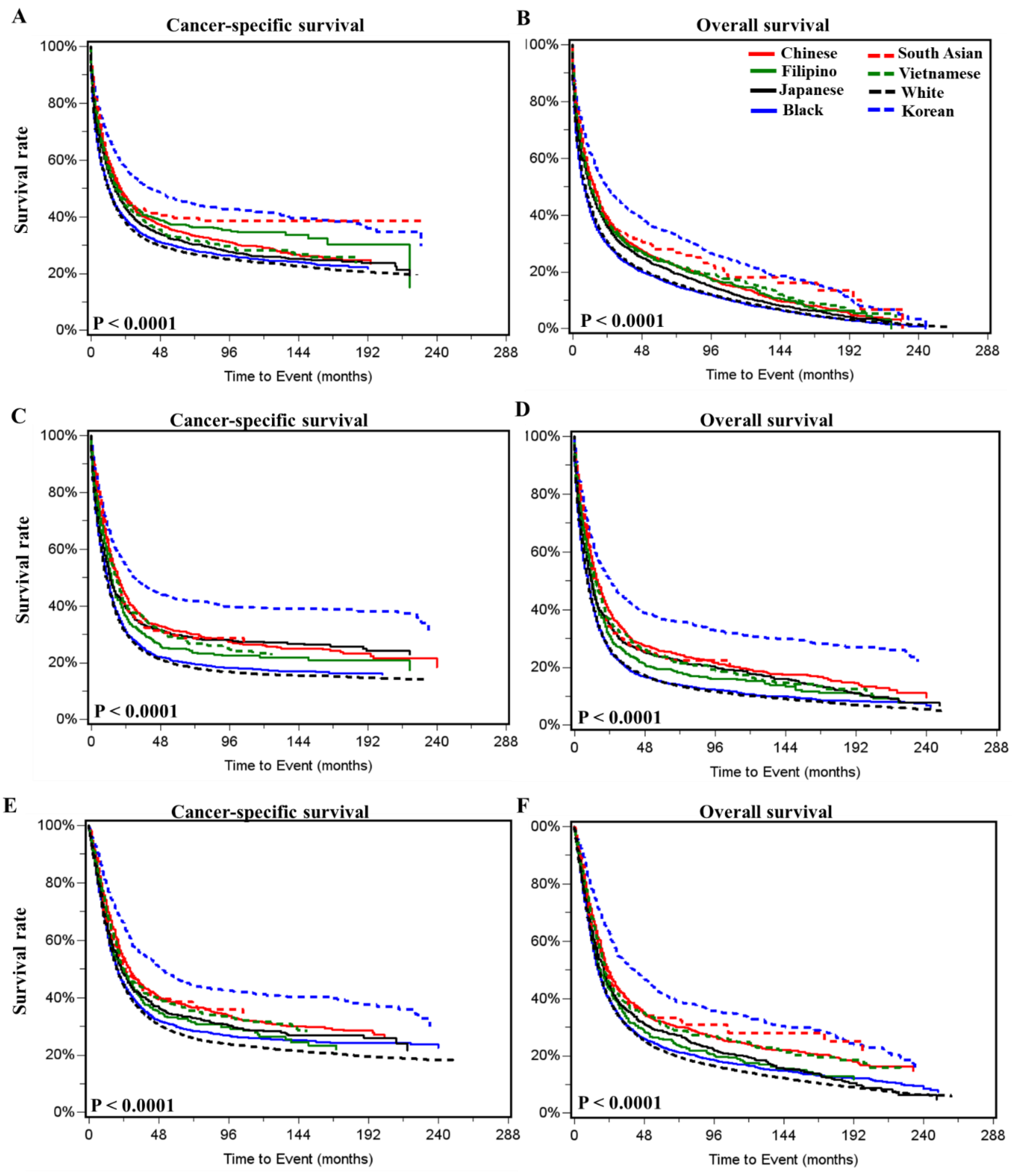
A
Cancer-specific survival
B
Overall survival
Chinese
Filipino
Japanese
Black
South Asian
Vietnamese
White
Korean
C
Cancer-specific survival
D
Overall survival
E
Cancer-specific survival
F
Overall survival
Survival rate
Time to Event (months)
P < 0.0001
0%
20%
40%
60%
80%
100%
0
48
96
144
192
240
288

Figure S5. Kaplan-Meier cancer-specific and overall survival curves among racial/ethnic groups of patients with gastric cancer after subgroup stratification. Cancer specific and overall survival curves are presented for patients with regional-stage tumors (**A and B, respectively**), patients with gastric cardia tumors (**C and D, respectively**), and those who received chemotherapy (**E and F, respectively**).

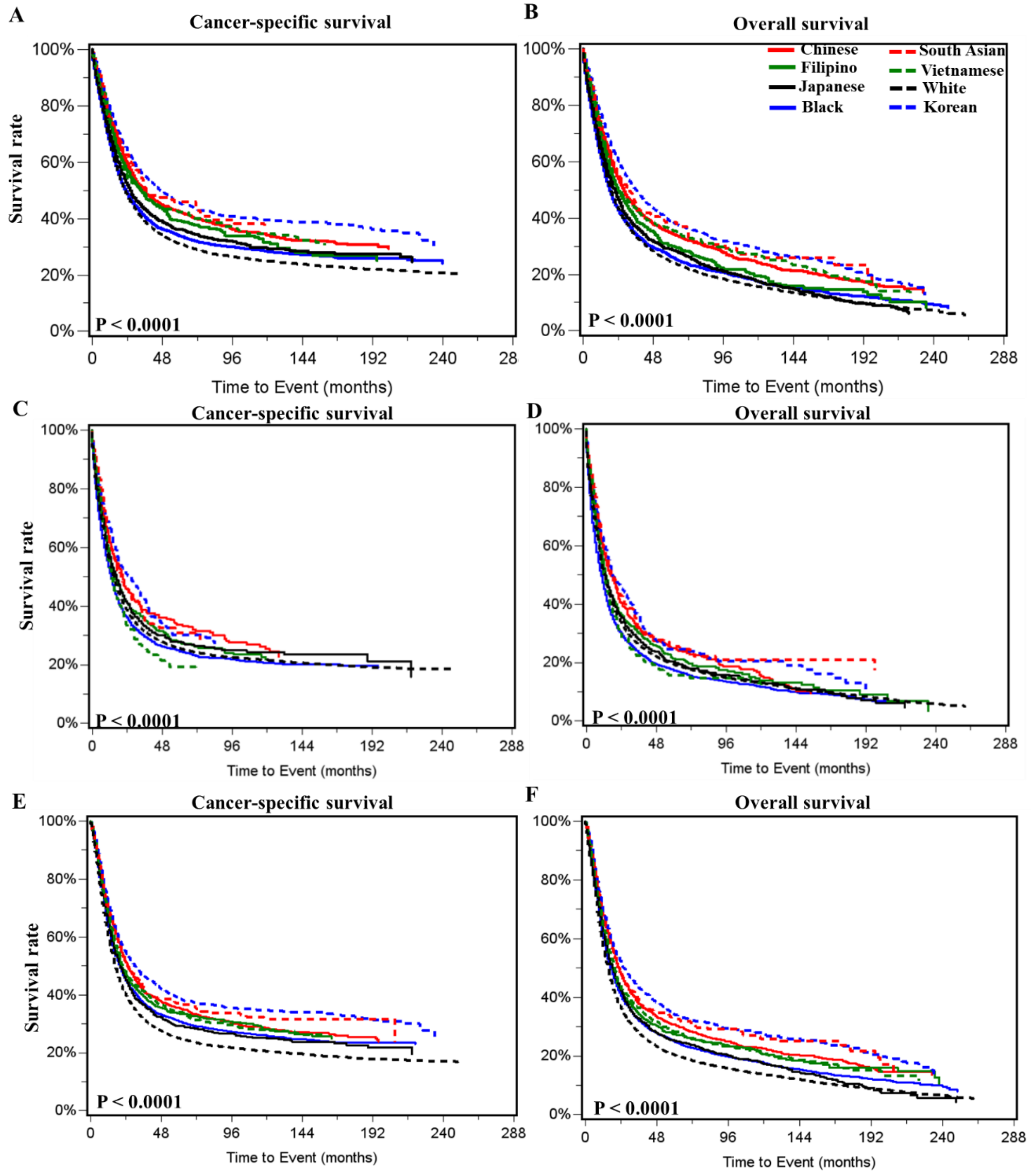
A
Cancer-specific survival
B
Overall survival
Chinese
Filipino
Japanese
Black
South Asian
Vietnamese
White
Korean
Survival rate
Time to Event (months)
P < 0.0001
C
Cancer-specific survival
D
Overall survival
E
Cancer-specific survival
F
Overall survival

Figure S6. Kaplan-Meier cancer-specific survival curves for gastric cancer patients by diagnosis period (2000-2010 vs 2011-2021). Curves are shown for Korean (**A**), Vietnamese (**B**), South Asian (**C**), White (**D**), Black (**E**), Chinese (**F**), Japanese (**G**), and Filipino (**H**) patients.

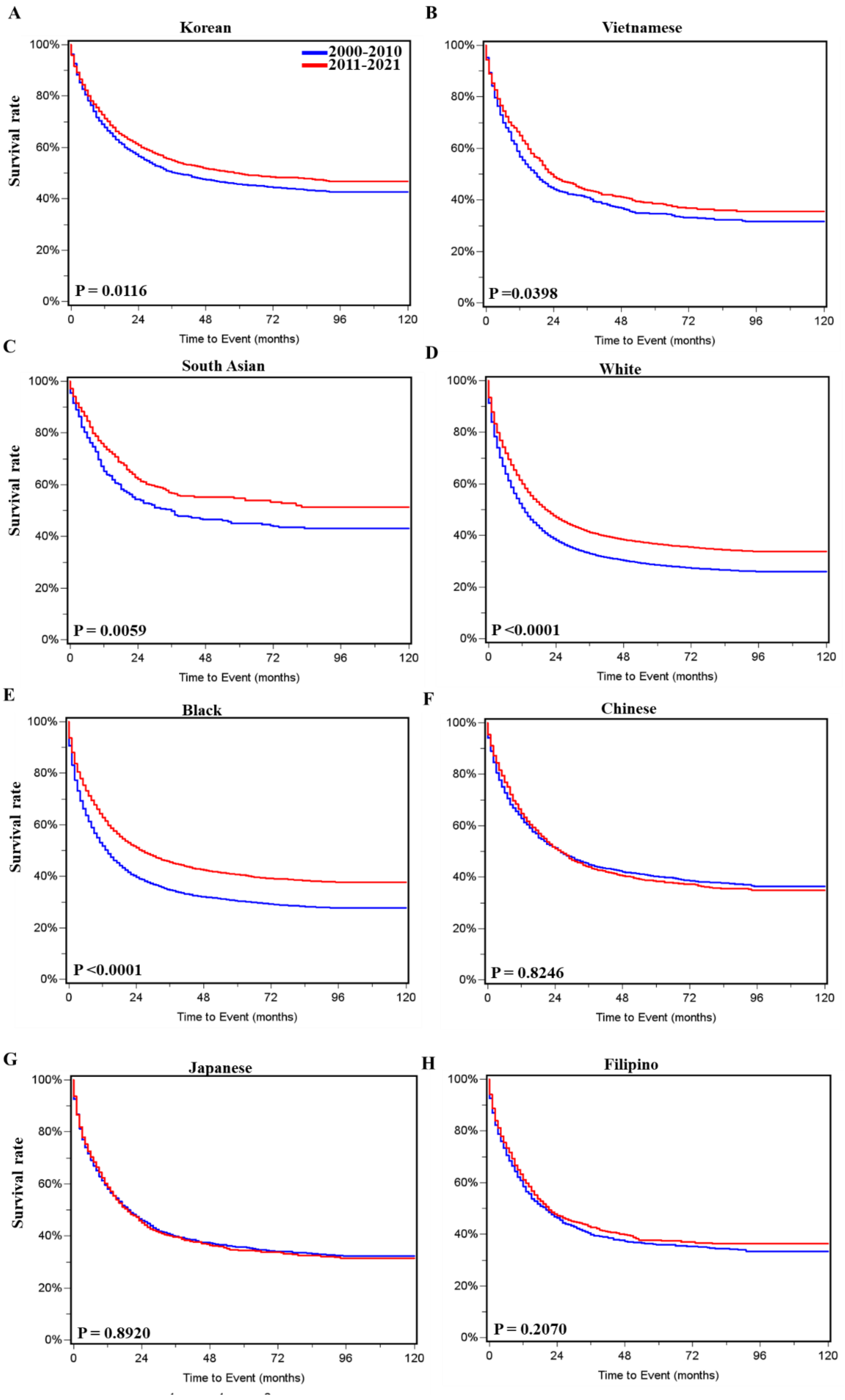
A
Korean
2000-2010
2011-2021
Survival rate
P = 0.0116
Time to Event (months)
B
Vietnamese
P =0.0398
Time to Event (months)
C
South Asian
Survival rate
P = 0.0059
Time to Event (months)
D
White
P <0.0001
Time to Event (months)
E
Black
Survival rate
P <0.0001
Time to Event (months)
F
Chinese
P = 0.8246
Time to Event (months)
G
Japanese
Survival rate
P = 0.8920
Time to Event (months)
H
Filipino
P = 0.2070
Time to Event (months)

Figure S7. Kaplan-Meier overall survival curves for gastric cancer patients by diagnosis period (2000-2010 vs 2011-2021). Curves are shown for Filipino (**A**), Vietnamese (**B**), South Asian (**C**), White (**D**), Black (**E**), Chinese (**F**), Korean (**G**), and Japanese (**H**) patients.

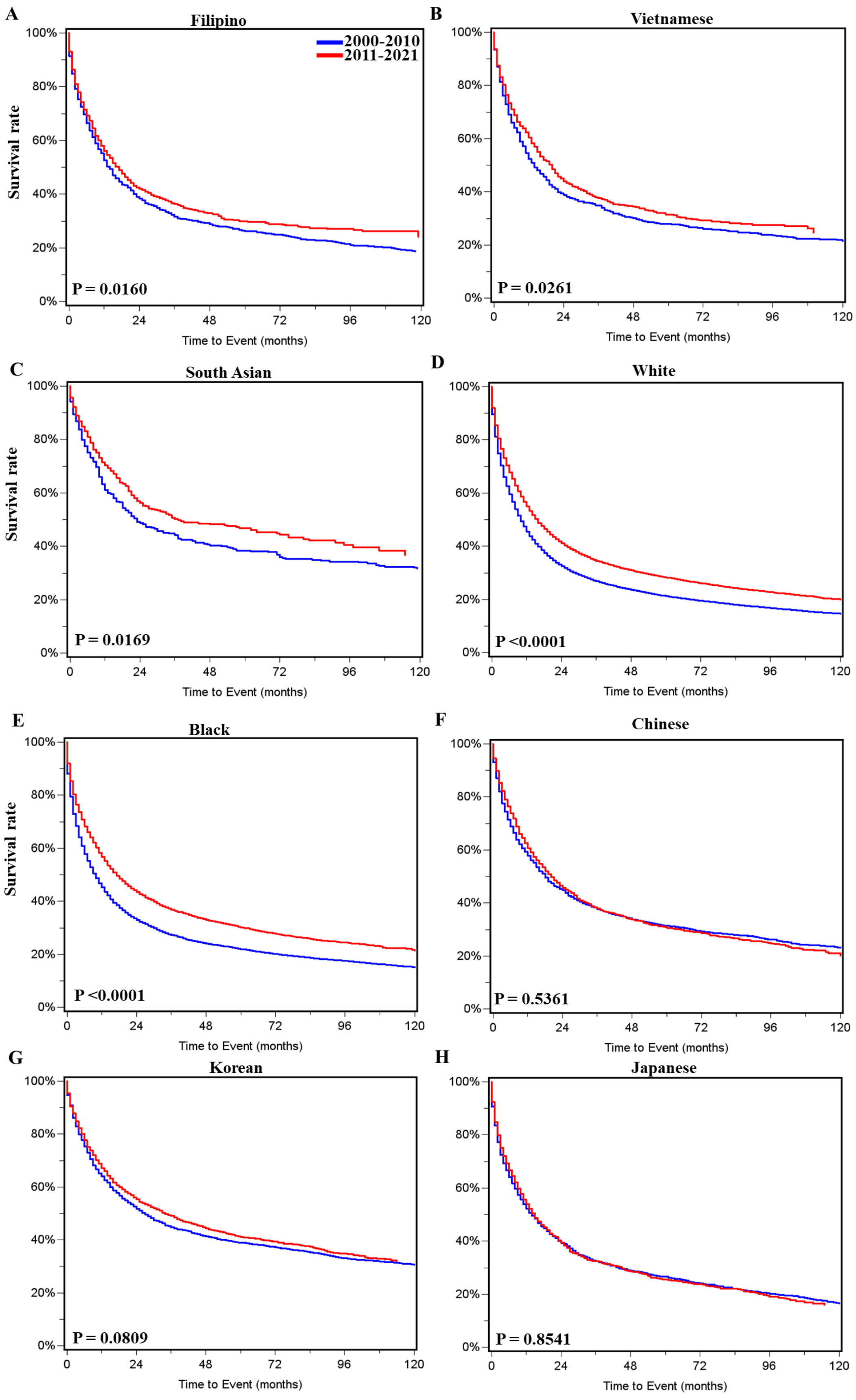
A
Filipino
2000-2010
2011-2021
Survival rate
100%
80%
60%
40%
20%
0%
P = 0.0160
0
24
48
72
96
120
Time to Event (months)
B
Vietnamese
P = 0.0261
C
South Asian
P = 0.0169
D
White
P <0.0001
E
Black
P <0.0001
F
Chinese
P = 0.5361
G
Korean
P = 0.0809
H
Japanese
P = 0.8541

Figure S8. **Demographic and clinical features of gastric cancer patients by ethnic group for the periods of 2000-2010 and 2011-2021. The proportions of cardia stomach cancer (A), Grade III/IV tumor (B), diffusive histological type (C), and radiotherapy (D).**

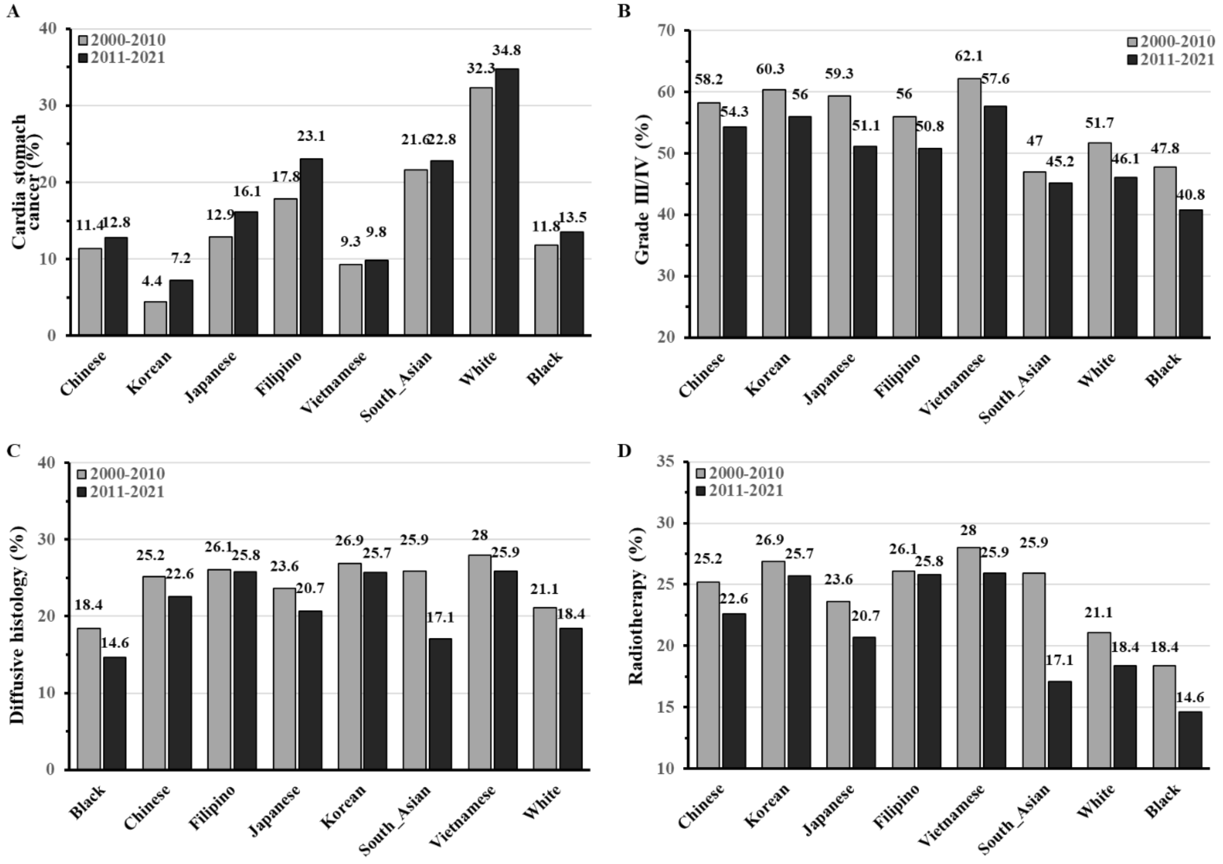

Supplement: Supplementary file 1 [file DataSheet1.pdf]
